# The carbon border adjustment mechanism is inefficient in addressing carbon leakage and results in unfair welfare losses

**DOI:** 10.1016/j.fmre.2023.02.026

**Published:** 2023-03-30

**Authors:** Xinlu Sun, Zhifu Mi, Lu Cheng, D'Maris Coffman, Yu Liu

**Affiliations:** a The Bartlett School of Sustainable Construction, University College London, London WC1E 7HB, United Kingdom; b College of Urban and Environmental Sciences, Peking University, Beijing 100871, China

**Keywords:** Carbon border adjustment mechanism, Carbon leakage, Climate change mitigation, Floor carbon price, Trade retaliation

## Abstract

The European Commission has proposed a Carbon Border Adjustment Mechanism (CBAM) to reduce carbon leakage and create a level playing field for its domestic products and imported goods. Nevertheless, the effectiveness of the proposal remains unclear, especially when it triggers threats of retaliation from trading partners of the European Union. We apply a Computable General Equilibrium model - Global Trade Analysis Project - to assess the economic and environmental impacts of different CBAM schemes. Here we show that the effectiveness of the CBAM to address carbon leakage risks is rather limited, and the CBAM raises concerns over global welfare costs, Correct to Gross Domestic Product (GDP) losses, and violation of equality principles. Trade retaliation leads to multiplied welfare losses, which would mostly be borne by poor countries. Our results question the carbon leakage reduction effect of a unilateral trade policy and suggest that climate change mitigation still needs to be performed within the framework of international cooperation.

## Highlights

• We found the carbon border adjustment mechanism reduces carbon leakage by 19%.
• Different policy schemes including carbon price and revenue usage are considered.
• Scenarios of cooperation and counteraction of trade partners are simulated.
• We revealed that the carbon border adjustment mechanism overburdens developing countries.

## Graphical abstract

**Figure:**
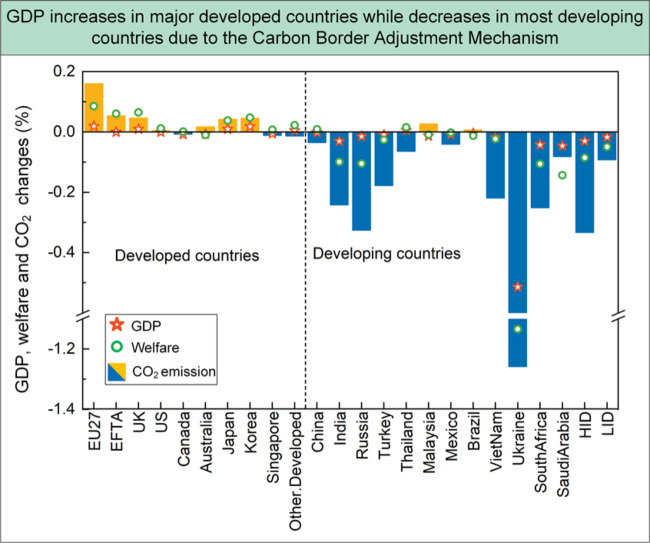

## 1 Introduction

The aim of a Carbon Border Adjustment Mechanism (CBAM) is to equalise the price of carbon between domestic products and imports with the objective of addressing the longstanding problem of carbon leakage. As global society is urgently demanding a green recovery from the COVID-19 pandemic, the European Commission published its proposal to introduce the CBAM, which is part of the “Fit for 55 package” to cut emissions by at least 55% by 2030. In Dec 2022, the European Council and Parliament reached a provisional deal on the CBAM. The CBAM is designed to function in parallel with the EU Emission Trading System (EU ETS) and to prevent carbon leakage. These goals are in accord with the ambition of the European Union (EU) as one of the main leaders in climate change mitigation in the world.

The CBAM helps to create a level playing field for the EU's domestically-produced and imported goods [1]. As the climate rules in the EU have become increasingly ambitious and strict, the carbon price in the EU ETS continues to increase due to the tightening emission quota. This increase leads to increased production costs for EU goods and gives a competitive advantage to products outside the EU that use carbon-intensive production processes [2,3]. This situation not only erodes the competitiveness of EU products but may also lead to a production relocation of carbon-intensive industries to regions without strong climate mitigation measures. The ensuing emission increase in non-abatement countries will undermine EU and global decarbonization efforts. To deal with carbon leakage risks, the European Commission intends to impose a carbon levy on imports from non-EU regions or to include imports in the EU ETS. Such a measure would see imported goods be hit with an environmental cost equivalent to that imposed when produced in the EU and thus protect the competitiveness of the EU's products. The CBAM is expected not only to help protect the abatement achievements of the EU's efforts but also to precipitate the climate actions of non-EU countries [4,5].

However, the potential of the CBAM to reduce global carbon leakage remains unclear, as its effectiveness is affected by various internal and external factors. The internal factors are related to policy design, including the scope of emissions, carbon price level, carbon intensity measurement and revenue usage. Firstly, the scope of emissions accounted in the CBAM is an important factor [6,7]. Researchers believe that for a better indication of the carbon contents of import goods and for more effective measurement, both direct carbon emissions during production and indirect carbon emissions from electricity usage and material production should be accounted for [4,8]. However, from a practical perspective, this inevitably causes immense challenges for data collection and logistical management. Secondly, the level of the carbon price directly influences the price changes of imported products and thus affects the effectiveness of the CBAM [9]. The future development of the EU ETS and the improvement of relevant policies will introduce uncertainties to the carbon price within the EU [10]. The average carbon price in the EU ETS was valued at $90.33/t in the last three months in 2021, while price level in the literature was apparently lower [11]. Thirdly, two ways of calculating carbon contents of the imported products are usually considered in modelling the effects of a CBAM. Practically, requiring the importers or foreign producers to report the facility-level emissions and activity data is preferable. But when modelling, one way is to use the average emission intensity in the export country to reflect the specific carbon content of the products from different origins. The defect of this approach is the complicated data collection and monitoring for the policy implementation. Another approach, using the EU production technology as the reference emissions, eliminates the overloaded administrative cost. Also, this will treat the imports from different countries identically and avoid discrimination on the imports [12]. Fourthly, the revenue usage of the CBAM influences the macroeconomic impact of the CBAM. A simulation comparing the revenue recycle and no recycling indicates that the macroeconomic gains by the CBAM revenue is much more than the direct trade interactions in the long run [13]. How the CBAM revenue will be used has not been clarified in detail, but most of them will be made into the EU budget as general income to support investments in green and digital transition [14,15].

The external factor that impacts the implementation of the CBAM includes the potential legal disputes and other countries’ responses. As the CBAM has the potential to enhance the climate ambition and facilitate the climate action outside the EU, it provides an opportunity for all nations to abate carbon emissions together. If other countries are willing to cooperate and implement more ambitious climate policy in response to the CBAM, for example, a global carbon tax or removing fossil subsidies [11], this would bring more carbon reduction benefits. However, cooperation is not necessarily guaranteed. Non-EU countries would interpret the carbon levy as a source of income for the EU's own fiscal budget and recognize the CBAM as protectionist [16], [17], [18]. For a more resilient transition to Net Zero, the EU passed the NextGenerationEU (NGEU) Recovery Plan in 2020 for economic recovery. In total, a stimulus package of over €2 trillion has been assigned by NGEU to rebuild a post-COVID-19 Europe [14] and the CBAM was proposed as a source of revenue to support NGEU investment [13]. Many countries have expressed their concerns about the CBAM. The United States warned the EU that the CBAM should be a “last resort” [19], while Australia criticized the CBAM for running “the risk of enhancing protectionism” and being “detrimental to global growth” [20]. Major developing countries protest against the CBAM because it shifts the economic burden of developed-world climate policies to the developing world through terms-of-trade effects and aims to protect of EU domestic production [21], [22], [23], [24]. The CBAM compels other countries to make the same efforts to decarbonize their economic structure and may be regarded as contravening the common but differentiated responsibility principle of the United Nations Framework Convention on Climate Change (UNFCCC) [25]. In addition, the CBAM may be charged with violating the General Agreement on Tariffs and Trade (GATT) because it favours domestic companies [26]. Other countries may therefore treat the CBAM as a carbon-levy trade barrier. Thus, the use of counter-tariffs by other countries may be inevitable and further cause uncertainty regarding the impacts of the CBAM [4].

Previous studies on the EU CBAM are insufficient to indicate the effectiveness of the CBAM because they did not consider all the factors discussed above [6,^27^,28]. Here, we quantitatively evaluate the economic and environmental impacts of the EU CBAM by comprehensively considering the internal and external factors, and the costs burdened by countries differing in income levels. Based on seven counterfactual scenarios, we simulate alternative CBAM schemes with different carbon prices, emission scopes and carbon intensity measurement, revenue usage and in particular, cooperation and retaliation by other countries. The simulation is conducted in a Global Trade Analysis Project (GTAP) Computable General Equilibrium (CGE) model with the latest data in 2014. In this study, the carbon intensity and technology of production of the initial equilibrium is used. The results show that the CBAM is a limited solution to address the carbon leakage problems caused by the EU ETS and brings scant carbon reduction effects to global carbon abatement. In addition, the CBAM will cause welfare losses and inequality in developing countries. Retaliation will weaken the carbon reduction effect and further lead to more than seven-fold global welfare losses, which will be borne by poor countries. However, cooperation between countries to promote floor carbon price brings much more carbon reduction gains and greatly mitigate carbon leakage risks. Clearly, the implementation of the CBAM must be based on effectively solving problems of justice and efficiency. This study indicates the insufficiency of the unilateral trade measurement in tackling carbon leakage risks and points out the necessity of international cooperation in climate change mitigation.

## 2 Materials and methods

### 2.1 The global trade analysis project model

A CGE model is typically utilized to address the impacts of trade policies. In particular, the GTAP model is a well-established and commonly used CGE model for evaluating the economic and environmental impacts of global trade policies [29]. It is a comparative static model that shows the differences between different possible states of the global economy [30]. The economic simulation of a policy shock is based on the optimal behaviour of several agents, namely, private households, governments, and companies. Consumers in the model aim to maximize their utility, while producers aim to maximize their profits and minimize their costs.

Products are categorized into two groups: energy commodities (i.e., coal, gas, crude oil, petroleum products, and electricity) and other commodities. All input sets are aggregated as two composite bundles of primary factors and intermediate inputs. Substitution of the input sets is structured by multi-level nested constant elasticity of substitution functions. The production of each sector is based on the assumption of constant returns to scale and a completely competitive market. In terms of international trade, commodities from domestic production and imports are treated as imperfect substitutes based on the Armington assumption [31]. Regional and national economies are connected by trade in commodities.

GTAP-E is the energy-environmental extension of the standard GTAP model, the most frequently employed extension of the standard GTAP model for research on carbon mitigation policies (i.e., carbon tax and emission trading) [32,33]. It focuses on a more precise structure of energy production and consumption, as well as the related carbon emissions (from fossil fuel combustion). The core idea is to consider substitution within and between fossil fuels, other types of energy sources, capital and labour. To do this, the model takes two steps to take energy commodities out of the intermediate input nest and incorporate them into the value-added nest [32]. First, energy commodities are separated into electricity and non-electricity groups including fossil fuels, where substitutions are allowed within and between the two groups. Then, the energy composite is combined with capital and other primary factors in a value-added-energy nest through a constant elasticity of substitution structure. The elasticity of substitution between capital and energy within the capital-energy composite nest (*σ_KE-inner_*) is usually smaller compared with the elasticity of substitution between the capital-energy composite and other primary factors (*σ_VAE_*), producing an overall negative substitution elasticity between capital and energy (*σ_KE-outer_*). The relationships are as follows:

(1) $$\sigma_{KE-outer}\;=\;(\sigma_{KE-inner}-\sigma_{VAE})/S_{KE}\;+\sigma_{VAE}/S_{VAE}$$

where *S_KE_* is the cost share of the capital-energy composite in the value-added-energy nest, and *S_VAE_* is the cost share of the value-added-energy composite in the output nest (see Fig. S1 for the production structure of GTAP-E).

Carbon emissions are calculated based on the energy consumption by firms, government, and private household. It is assumed that emissions are proportional to energy consumption [33], for example:

(2) gco2fd(e,i,r)=qfd(e,i,r)

where *gco2fd* is changes of emissions from firms’ domestic energy consumption; *qfd* is the changes of firms’ domestic energy consumption; *e* represents energy type, including coal, oil, gas, and oil products; *i* represents industrial sector; *r* represents country.

The latest GTAP model data, version 10, is employed in this study, and the reference year in GTAP 10 is 2014, which is the latest year available. Although it might be better to update the data to the actual year of CBAM implementation, the economic effect of the COVID-19 pandemic would cause profound uncertainty in updating and projecting the data. Therefore, this study conducted a counterfactual analysis based on analysing the 2014 data. In GTAP 10, 141 countries and 65 sectors are included. In this study, the 141 countries are aggregated into 26 countries/regions, and the 65 sectors are aggregated into 17 sectors (Table S1). Based on data aggregation, the five types of energy products are separated into five individual sectors to better simulate the trade flows in the energy market and the carbon emission changes of different types of energy goods. In addition, the five primary factors in production are considered in GTAP-E, namely, land, capital, natural resources, skilled labour, and unskilled labour. Limitations exist in this study due to data availability (for example, failure to reflect the impact of energy prices change in 2022 and the war in Ukraine), and the assumptions of the CGE model (for example perfect information, rational behaviour, etc.), the study enables the assessment of long-term impact of the CBAM. The results help to compare the effectiveness of various CBAM schemes to reduce carbon leakage caused by the EU ETS. To test the robustness of the results, we conduct a sensitivity analysis with respect to the Armington elasticity and substitution elasticity of capital-energy composite to show how changes in parameters in the CGE model will affect the robustness of our results (see supplemental materials for details).

### 2.2 Scenarios of different policy schemes

As a pre-simulation scenario, the current carbon prices are implemented in the existing carbon markets, for example, a carbon price at $90/t is implemented in the EU (Table S2 for carbon prices of other countries). Carbon leakage rate is calculated as the proportion of increased carbon emissions outside the regions with the corresponding carbon price to the reduced carbon emissions in the regions.

In designing different scenarios regarding possible CBAM schemes, it is necessary to consider the following issues: the sectors and countries to which the CBAM applies, carbon accounting methods, carbon price and the referred technology of the production process. Five scenarios simulating different CBAM schemes are set in this study: a benchmark scenario, D90, with direct (scope 1) emissions accounted for and an average carbon price level in line with the EU ETS; an indirect emission (scope 1 and 2 emissions) scenario, E90; a higher carbon price scenario, D200; a scenario, D90_EUintst, with EU's production carbon intensity rather than the local production carbon intensity applied for tariff calculation; and scenario D90_otax simulates the impact of the CBAM revenue usage, where output tax is reduced in the EU by recycling the CBAM revenue. Before simulating the impacts of the CBAM, a baseline scenario is set by implementing the carbon prices of the current carbon markets in the EU and other ETS. The carbon prices in these countries are set as the average level in 2021 or in the last month in 2021 when carbon price increased dramatically (Table S2). Carbon prices in non-EU countries are adjusted according to the emission coverage ratio compared with the EU ETS [11]. The CBAM schemes under all scenarios apply to all sectors covered in the EU ETS. Currently, the European council and Parliament agree on several carbon-intensive products at a high level of carbon leakage risk that are covered by the CBAM: iron and steel, cement, fertilizer, aluminium, hydrogen, and electricity generation, with the goal to include all EU ETS sectors by 2030. To better evaluate the impact of the CBAM, the complete list of sectors in the EU ETS is considered in the simulations (see carbon-intensive sectors plus oil products and electricity in the energy sectors in Table S1).

Both the direct emissions and embodied emissions (plus indirect emissions) of import products are considered to determine the scope of emission accounting. According to the proposal of the provisional deal of the European Council and Parliament, the CBAM will apply to direct emissions for some products and both types of emissions for the other. Indirect emissions from electricity utilization during production of cement, fertilizer and power will also be covered, which will improve policy efficiency and mirror the scope of the EU ETS. The literature on the impacts of the CBAM agrees that both the direct and indirect carbon emissions of import products should be considered to ensure the effectiveness of the CBAM [4]. However, they also admit that accounting for direct carbon emissions in the CBAM would be more practical due to a lack of technical and data support to measure embodied carbon emissions [25]. To deal with data availability, some scholars have suggested that a benchmark carbon intensity representing the average performance of a sector may be feasible [7], for example, using the EU carbon intensity to calculate carbon content in the EU's imports. However, this approach fails to measure the emissions of an individual region [25]. Others have proposed that indirect emissions mainly come from electricity usage, and they have measured embodied emissions based on the proportion of electricity usage and the total emissions of the electricity sector [34]. In this study, the benchmark scenario (D90) accounts for only direct emissions and scenario E90 employs the embodied carbon emissions of imports to explore the impact of the emission scope on CBAM effectiveness. Both scenarios measure carbon content according to the local production process. And based on D90, the scenario D90_EUintst using the EU's production carbon intensity is designed.

We calculate the direct carbon emissions of import products based on the technologies in exporting countries and the indirect carbon emissions due to electricity usage in the production process, which can better reflect the actual carbon content in trade goods. The calculation is as the following Eq. 3 and Eq. 4 [35]:

(3) $$\begin{array}{c} E_{\text{dirt},r}=C_{r}\;/\;VOM_{r} \end{array}$$

(4) $$\begin{array}{c} E_{\text{emb},r}=\left(C_{r}\;+\;VDFM_{\text{power},r}\cdot \;c_{\text{power},r}\;/\;\mathrm{vo}m_{\text{power},r}\right)\;/VOM_{r} \end{array}$$

where ***E***_dirt,r_ is the vector of the direct emission intensity in country r, ***C***_r_ is the vector of the sectoral carbon emissions from fossil fuel, and the elements of vector ***VOM***_r_ are the values of the sectoral total output in country r. The embodied emission intensity ***E***_emb,r_ in country r is the direct emission intensity plus indirect emission intensity, where ***VDFM***_power,r_ is the consumption of electricity by each sector in country r, *c*_power,r_ and *vom*_power,r_ are the carbon emission and total output of the electricity sector in country r, and the two variables are elements of the vector ***C***_r_ and ***VOM***_r_, respectively.

The carbon price may greatly affect the effectiveness of the CBAM policy. According to the CBAM proposal of the European Commission, the carbon price of the CBAM will not exceed that of the EU ETS to avoid any violation of GATT principles [14]. Thus, the price in the GTAP model, US$90.33/t, is set as the carbon price under basic scenario D90, which is the average price in the EU ETS in the last month in 2021. As the carbon cap will be further constrained in the future, the carbon price will increase due to the decrease in permits. Thus, scenario D200 simulates the impact of a carbon price increase from $90/t to $200/t. The carbon pricing in the CBAM finally produce an increase in the tariff rate of the imported products to the EU. Tariff shocks (change power) of the EU's imports are calculated via Eqs. 5-7:

(5) $$\begin{array}{c} \mathrm{VXCO}2_{i,r,\text{EU}}\;=\;\left(p_{\mathrm{CBAM}}\;-\;p_{r}\right)\;\times e_{i,r}\;\times \mathrm{VXM}D_{i,r,\text{EU}} \end{array}$$

(6) $$\begin{array}{c} \mathrm{tm}s_{i,r}=\frac{\mathrm{VIM}S_{\;i,r,\text{EU}}}{\mathrm{VIW}S_{\;i,r,\text{EU}}} \end{array}$$

(7) $$\begin{array}{ccc} \Delta \text{tm}s_{i,r} & = & \frac{\text{tm}s_{i,r}^{{}^{\text{'}}}-\text{tm}s_{i,r}}{\text{tm}s_{i,r}}=\frac{\frac{\text{VIM}S_{i,r,\text{EU}}+\text{VXCO}2_{i,r,\text{EU}}}{\text{VIW}S_{i,r,\text{EU}}}-\frac{\text{VIM}S_{i,r,\text{EU}}}{\text{VIW}S_{i,r,\text{EU}}}}{\frac{\text{VIM}S_{i,r,\text{EU}}}{\text{VIW}S_{i,r,\text{EU}}}}\\ & = & \left(p_{\text{CBAM}}\;-\;p_{r}\right)\;\times e_{i,r}\;\times \frac{\text{VXM}D_{i,r,\text{EU}}}{\text{VIM}S_{i,r,\text{EU}}} \end{array}$$

where Eq. 5 calculates the value of carbon tariff paid for the export i from country r to the EU (*VXCO2*_i,r,EU_), Eq. 6 is the equation measuring tariff *tms*_i,___r__ in the GTAP, Eq. 7 measures the tariff change power Δ*tms*_i,___r__ of the export i from country r to the EU induced by an ad valorem carbon tax. *p*_CBAM_ is the carbon price in the CBAM scheme, the unit of which is $ per ton carbon dioxide emissions, and *p_r_* is the domestic carbon price in country r (Table S2); *e*_i,r_ is the carbon intensity of product i made in country r, i.e., the emissions contained per unit of exported product i; *VXMD*_i,r,EU_ is the value of export i from country r to the EU at the market price; *VIMS*_i,r,EU_ and *VIWS*_i,r,EU_ is the value of import i from country r to the EU at EU's market price and world price, respectively. Additionally, *e*_i,r_ is an element of vector ***E***_dirt,r_ or ***E***_emb,r_.

As the proposed scheme of the CBAM by the Europe Commission, the European Free Trade Association (EFTA) countries will be exempted for the CBAM in this study. The amendments adopted by the European Parliament emphasized the technical and financial support for decarbonization in the Less Developed Countries (LDC) countries. In this study, LDC countries are also exempted. An aggregated region named LDC contains the less developed countries defined by the United Nations [36] (Table S3). Note that some of the less developed countries on the UN's list are aggregated in the “rest of the world (ROW)”. Therefore, exemptions are offered to EFTA, LDC, and the ROW under each scenario.

### 2.3 Scenarios of abatement cooperation and trade retaliation

Furthermore, in consideration of the efficiency, justice, and legal issues of CBAM design, we set two scenarios to simulate the impact of different responses of non-EU countries, including international cooperation and trade retaliation by other countries. The scenario Cooperation assumes that global cooperation is agreed on and the floor carbon prices is implemented where carbon prices are determined according to the development level of the countries. Based on the IMF's proposal for a floor carbon price scheme, the developed countries agree on a carbon price at $75/t, while high-income and low-income developing countries have less carbon price at $50/t and $25/t [37]. As the carbon price in the EU ETS already exceeds the price in this scheme, here, we assume that the developed countries implemented a carbon price the same as in the EU ($90/t), while higher- and lower-income developing countries (HID and LID, respectively) have carbon prices at $60/t and $30/t, respectively. The impact of both the higher carbon prices in these countries and the CBAM charged for imports from the developing countries are simulated in the scenario Cooperation.

By contrast to possible cooperation, many other countries, especially developing countries, may claim that the CBAM is trade protectionism based on environmental trade barriers. They have expressed their concerns about and strong opposition to any type of trade measure that would impose carbon costs on their exports [16,^19^,^20^,^22^,23]. Previous conflicts on the EU's proposal to include international aviation into the EU ETS confirm the potential for trade disputes induced by the CBAM [38]. Non-EU countries may retaliate against the CBAM by imposing a tariff on EU products.

The scenario Retaliation is designed where the EU's major trade partners retaliate on the CBAM. As the trade dispute resolution process of the World Trade Organization (WTO) incurs great costs, it is suggested that major economies will tend to choose trade retaliation, while small countries will not. Thus, under scenario Retaliation, we investigated the major trade partners of the EU and the sensitive sectors in the trade dispute between these countries. We combed through trade dispute records from 1990 to 2019 in the World Bank Temporary Trade Barriers Database and identified the sectors most involved in trade reactions to EU imports. In designing the trade retaliation under scenario Retaliation, we referred to these major trade partners and corresponding sectors to determine the countries initiating countermeasures and the targeted sectors (Table S4). The tariff changes were determined based on export losses, which means that trade retaliation aims to create the same export losses for the EU as the retaliating country experienced. Specifically, tariff changes are first determined by the value of tariff burden experienced by the raiser country, calculated by Eq. 8. Then, an extra tariff shock beyond scenario D90 is determined by the GTAP model, and the export losses for the EU are estimated. The tariff is then adjusted until the export losses of the EU and the raiser country are equal.

(8) $$\begin{array}{c} \Delta \text{tm}s_{j,\text{EU},r}=\frac{\sum_{i}\text{VXCO}2_{i,r,\text{EU}}\;}{\text{VIM}S_{j,\text{EU},r}} \end{array}$$

where Δ*tms*_j,EU,r_ is the initial tariff change power of the imports j from the EU to the raiser country r; *VIMS*_i,EU,r,_ is the value of import j from the EU to country r at the market price. In this way, the change in the retaliatory tariff is determined to compensate for export loss due to the CBAM.

### 2.4 Gini coefficient

The Gini coefficient, proposed by the Italian statistician and socialist Corrado Gini, is a widely-used statistic to measure inequality of income and wealth [39]. A Gini coefficient of zero represents the perfect equality, indicating people all have the same income or wealth. In contrast, a Gini coefficient of one delineates absolute inequality, meaning one person has all the income or wealth whereas other people have none. We calculated Gini coefficients to evaluate the impact of CBAM on inequality. The following Eq. 9 is the calculation of the Gini coefficient in this study:

(9) $$G\;=\sum_{i=1}^{26}\text{Po}p_{i}\text{Inc}m_{i}+2\sum_{i=1}^{26}\text{Po}p_{i}(1-T\_\text{inc}m_{i})-1$$

where G is the Gini coefficient; $Pop_{i}$ is the ratio of population in country i to total population in the world, $Incm_{i}$ is the ratio of income in country i to the total income in the world; $T\_incm_{i}$ is the cumulative proportion of income in country i, and i denotes the number of country/region (*i* = 1, 2, 3, …, 26).

Similarly, by replacing the income-related statistics to carbon emissions, we also calculated the carbon emission Gini coefficient (C-Gini) [40]. The Gini coefficient and C-Gini coefficients are 0.60 and 0.45 when current carbon prices are implemented, indicating carbon inequality is relatively moderate compared with income inequaility [40].

### 2.5 Data sources

The trade, economic, population and carbon emission data are accessed via the GTAP database (https://www.gtap.agecon.purdue.edu) for the basic CGE modelling. The trade barrier data, used to determine the sensitive sectors in the historical trade countermeasures, are from the World Bank Temporary Trade Barriers Database (https://www.worldbank.org/en/data/interactive/2021/03/02/temporary-trade-barriers-database). The latest carbon price in the EU ETS is provided by International Carbon Action Partnership Allowance Price Explorer (https://icapcarbonaction.com/en/ets-prices). Carbon prices of other countries are from the World Bank carbon dashboard (https://carbonpricingdashboard.worldbank.org/). Developing and developed countries are classified according to the World Economic Outlook database by International Monetary Fund [41] (https://www.imf.org/external/pubs/ft/weo/2015/02/weodata/groups.htm#cc). Categories of the developing countries into high- and low-income groups are from the World Bank country classification [42]. National consumption-based carbon emissions are available from Global Carbon Budget [43] (https://www.globalcarbonproject.org/carbonbudget/21/data.htm).

## 3 Results

### 3.1 Limited carbon leakage reduction effects are found

The carbon leakage rate caused by the current carbon price ($90/t) EU ETS, measured by the ratio of increased emissions in non-EU regions to the reduced emissions in the EU and EFTA regions, is about 20.2% (Table 1). Scenario ETS, where the current carbon prices in the EU and other ETS are implemented to the corresponding carbon markets, shows that the EU ETS leads to about 588.9 Mt carbon emission reduction in the EU and EFTA countries, while carbon emissions increase by 119.1 Mt in the non-EU countries. The reduced carbon emissions are higher than in the literature [44], because the carbon price implemented in this study is the latest level and much higher than the history level. This carbon leakage rate is consistent with results in the literature [45]. Carbon prices in other developed countries like the UK, US, Canada etc., lead to reduced carbon emissions by 401.8 Mt and emission leakage by 25.4 Mt. Carbon leakage rate of current carbon prices in these developed countries is 6.3%. Carbon prices in developing countries, such as China, Mexico, and South Africa, can reduce carbon emissions by 658.4 Mt accompanied with a carbon leakage rate of 1.3%.

**Table 1:** **Economic and environmental impacts of domestic carbon prices in EU ETS and other countries**.

	Carbon reduction	Emission leakage	Leakage rate	Welfare	Real income loss	Real GDP loss
	Mt	Mt	%	Billion $	%	%
**ETS**						
**At current carbon prices**	−1609.2	86.8	5.4	−108.7	0.16	0.14
EU ETS	−588.9	119.1	20.2	−86.2	0.13	0.11
ETS of other developed countries	−401.8	25.4	6.3	−15.9	0.02	0.02
ETS of developing countries	−685.4	9.2	1.3	−6.5	0.01	0.01
**D200**						
**EU ETS - 200$/t**	−961.8	228.0	23.7	−196.4	0.29	0.25
Price increase from 90 to 200$/t	−372.9	108.9	29.2	−110.2	0.16	0.14
**Cooperation**						
Total effects of floor carbon prices	−7960.1	44.0	0.6	−318.8	0.47	0.41
Developed - 90$/t	−3372.1	295.0	0.9	−125.1	0.18	0.16
Higher-income developing - 60$/t	−4187.2	158.7	3.8	−95.8	0.14	0.13
Lower-income developing - 30$/t	−850.4	40.0	4.7	−7.2	0.01	0.01

Notes: The baseline is the initial equilibrium where no carbon prices and CBAM exist. Scenario ETS simulates the effects of the carbon prices in the current emission trading systems in different regions. The implemented carbon prices and emission trading systems can be found in Table S2. Scenario D200 simulates a domestic carbon price at $200/t only in EU and the EFTA countries. Scenario Cooperation simulates the effects of floor carbon prices in three country groups, where developed countries, excluding the EU and EFTA, have a carbon price at $90/t, the same as the EU ETS; higher-income developing countries have a moderate carbon price at $60/t; lower-income developing countries have a low carbon price at $30/t. The column carbon reduction means reduced carbon emission in the regions where the corresponding carbon price is implemented, and emission leakage is the increased carbon emissions outside the regions with carbon price. Leakage rate is the ratio of emission leakage to carbon reduction.

Although the cost-benefit efficiency of the CBAM should be affirmed, the overall environmental benefits of the CBAM are insufficient to address the carbon leakage caused by the EU ETS. In the benchmark scenario D90, the emission reduction in non-EU countries is 22.5 Mt, accounting for only 18.9% of the carbon emission leakage caused by the EU ETS (Table 2). Such emission reduction gains are offset by 4.9 Mt emission increase in the EU and exemption areas. Consequently, total carbon emission reduction globally is 17.4 Mt. Regarding the economic impacts, it is affirmable that the CBAM is effective. The global welfare losses (measured by equivalent variation) are approximately $0.67 billion, accounting for 1% of the welfare loss of the EU ETS. This indicates that the CBAM causes an extra 1% economic loss but addresses 18.9% of the carbon leakage of the EU ETS.

**Table 2:** **Economic and environmental impacts of different policy schemes and responses at the corresponding carbon prices**.

	CO_2_ in non-EU	Emission leakage reduction	CO_2_ in EU and exempted areas	Welfare	Real income loss	Real GDP loss	C-Gini change
	Mt	%	Mt	Billion $	%	%	%
**ETS. At current carbon prices**							
D90	−22.5	18.9	4.9	−0.67	0.001	0.001	0.019
E90	−29.7	24.9	6.7	−0.78	0.001	0.001	0.019
D90_Euinten	−10.5	8.8	1.6	−0.59	0.001	0.001	0.002
D90_otax	−22.3	18.7	5.5	−0.30	0.000	0.000	0.019
Retaliation	−19.6	16.5	1.9	−4.58	0.006	0.005	0.022
**D200. EU ETS at 200$/t**							
D200	−36.4	16.0	8.1	−2.3	0.003	0.003	0.022
**Cooperation. Floor carbon prices**							
Cooperation	−10.3	8.6	2.9	−0.05	0.000	0.000	0.012

Notes: the emission and economic impacts of the CBAM scenarios in this table are the variation compared with equilibrium at the corresponding carbon prices in Table 1. Emission leakage reduction is the ratio of carbon reduction in non-EU countries (excluding EFTA, LDC, and ROW) to emission leakage due to carbon prices in the EU and EFTA. D90 is the scenario in which the CBAM accounts for direct carbon emissions and sets a carbon price of $90/t. E90 is the scenario with indirect emissions (scope 1 and 2 emissions) accounted for and a carbon price at $90/t. D90_EUintst is the scenario with the policy scheme of D90 but use carbon intensity of the EU production processes to calculate CBAM tariff changes. D90_otax simulates the impact of the CBAM revenue usage by reducing the output tax to compensate for the price change in the EU. Scenario Retaliation simulates the non-cooperation responses of countries faced with a CBAM, in which the main trade partners of EU raise trade retaliation and impose tariffs on anti-dumping products based on historical trade disputes. D200 is the scenario with direct emissions accounted for and a carbon price at $200/t. Scenario Cooperation simulates a global cooperation scheme where countries agree on global floor carbon prices, and EU implements the CBAM to the countries with lower carbon prices than the price in EU ETS.

**Fig. 1:**
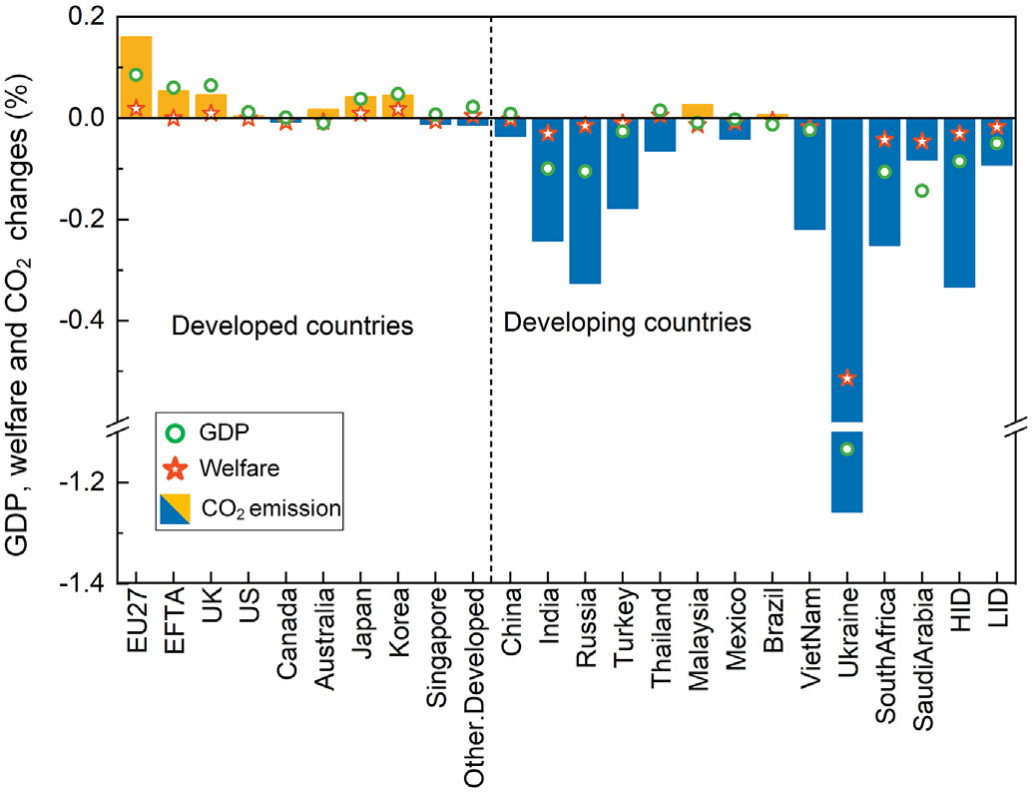
**Changes in GDP, carbon emissions and welfare due to a carbon border adjustment mechanism**. This scenario (D90) refers to a CBAM with direct carbon emissions accounted for and a carbon price set to $90/t. HID denotes high-income developing countries and LID denotes low-income developing countries.

**Fig. 2:**
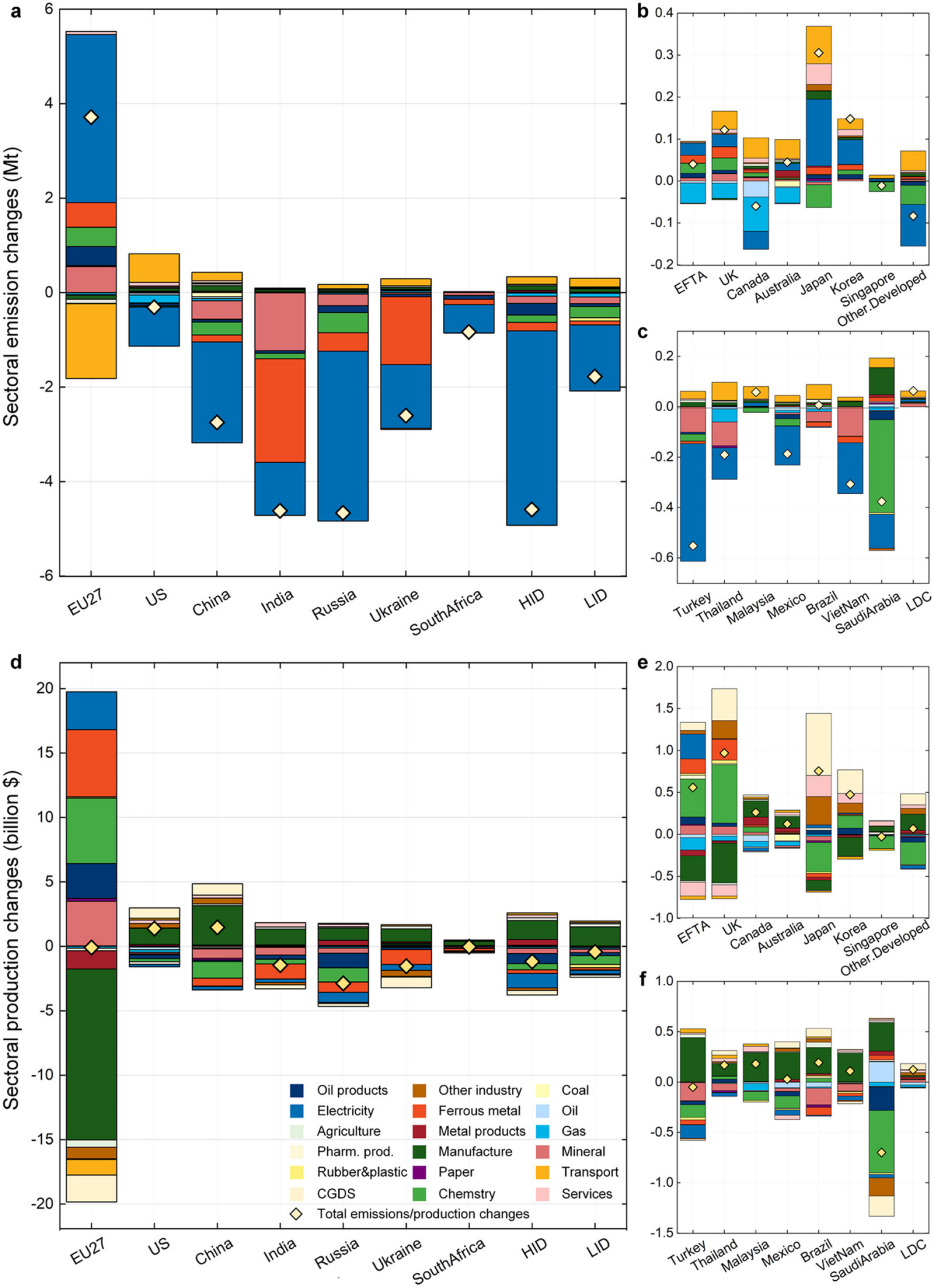
**Impacts on the carbon emissions and production of 17 sectors.** This scenario (D90) refers to a CBAM with direct carbon emissions accounted for and a carbon price set to $90/t. (a) Sectoral emission changes of the major impacted countries. (b) Sectoral emission changes of developed countries. (c) Sectoral emission changes of developing countries. (d) Sectoral production changes of the major impacted countries. (e) Sectoral production changes of developed countries. (f) Sectoral production changes of developing countries.

A clear impact on equality is seen in the modelling results. Carbon emission reductions created by the CBAM mainly occur in developing countries, namely, Russia, India, Turkey, South Africa, Ukraine, etc., (Fig. 1), resulting from a shrinkage in production and exports in these developing countries. Regarding economic impacts, GDP increases in most developed countries. The EU enjoys the largest GDP increase (0.1%) due to the CBAM. In contrast, developing countries, e.g., India, Russia, Ukraine, and South Africa, experience a GDP decline due to the CBAM. On the other hand, the EU is the major beneficiary, enjoying a welfare increase of $2.6 billion, accounting for 73% of the total welfare gains due to the CBAM. Ukraine experiences the most economic loss in this analysis because of its export loss in ferrous metal industry. While the value of production in ferrous metal makes it one of the most important sectors in Ukraine amongst the secondary industry, it is also amongst the most carbon-intensive sectors and suffers greatly from the CBAM (Fig. 2). With price increase and demand decline in the EU market after the implementation of the CBAM, exports of ferrous metal products from Ukraine to the EU are decreased by about 34%. Although increase in ferrous metal exports to other countries offsets some of the negative impacts, the CBAM finally leads to ferrous metal production in Ukraine declining by almost 6%. At the same time, the declined household income in Ukraine leads to depressed consumption by private households, especially in the service sector (accounting for more than half of the private consumption decrease), which is sensitive to income levels. Malaysia and Brazil are the only two developing countries where the carbon emissions increase. These two countries benefit from the relatively low carbon intensity of the domestic production and therefore experience lower tariff impacts (Fig. S2). Chemistry in Malaysia is the most affected sector because of the CBAM (Fig. 2). The shrunken demand in carbon-intensive products releases the demand of primary factors such as labour and capital in the developing countries, and therefore decreases the price of primary factors. Such changes enhance the competitiveness of manufacturing goods and low-carbon manufacturing production rises. Export of manufacturing goods from Malaysia to the developed countries increases and leads to increased demand in transportation, and therefore increased carbon emissions.

Carbon emission reduction is mainly attributed to reduced production in carbon-intensive industries in developing countries with China, India, Russia, Ukraine, and South Africa seeing a major impact in this respect. The production of energy-intensive industries experiences a relocation from developing countries to developed countries. Due to the increased import price of energy-intensive goods in the EU, the boosted domestic production of such products leads to a carbon emission rise in the electricity, metal, chemical, and mineral sectors. Although carbon emissions in most sectors in the EU increase as the CBAM protect the comparativeness of domestic products, there is a significant emission reduction in the transport sector because of a shrinkage in the usage of oil products (Fig. 2 and supplementary data). Such reduced oil products usage is resulted from both decreased demand of transport products and price increase of oil products in the EU, while some reduction is substituted by increased gas usage due to the lower prices of gas goods. Outside of the EU, the production of ferrous metals and chemical products increases in developed countries such as the EFTA and the UK, and decreases in developing countries such as China, India, and Russia. Such production shrinkages cause emission reductions mainly in the electricity, metal, and mineral sectors in developing countries. The trends of production relocation put pressure on developing countries to upgrade industrial structure for green and sustainable development.

The carbon tariff on the carbon-intensive products shows a significant impact on other sectors, especially manufacturing. In many developed countries, like the EU, EFTA, the UK, Japan, and Korea, production in manufacturing drops off, while in other countries, manufacturing production is boosted. In developing countries, such as India, the boosted manufacturing production is mainly driven from enlarged exports (supplementary data). This is because the stricken carbon-intensive production in developing countries, suffering as a result of the CBAM, weakens the competitiveness of related sectors, and therefore the declined production releases primary factor demand and decreases primary factor prices. Furthermore, the manufacturing sector in developing countries benefits from this and subsequently gains a comparative advantage in global markets. The increased price of the imported raw material and therefore increased production cost are the main reasons of the contracted downstream manufacturing in the EU. In other developed countries, the declined manufacturing production is because of the drop in the export of manufacturing products.

**Fig. 3:**
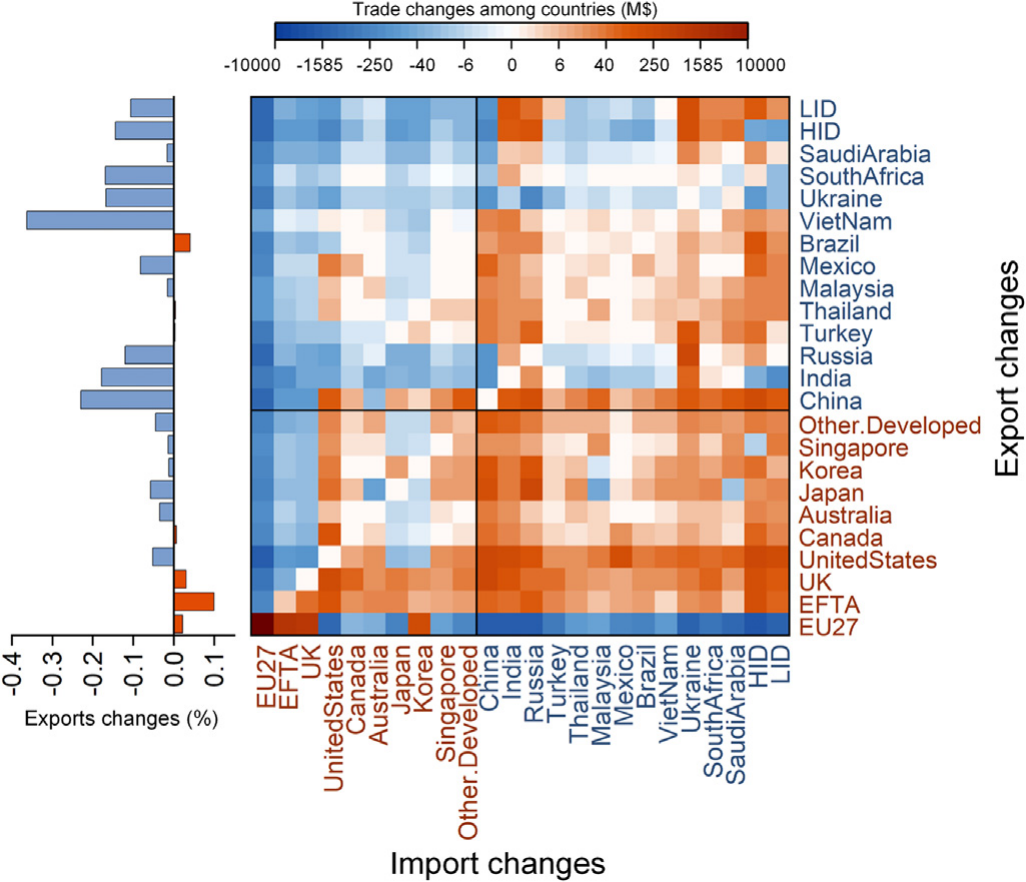
**Impacts on exports and imports of countries and regions.** This scenario (D90) refers to a CBAM with direct carbon emissions accounted for and a carbon price set to $90/t.

Overall, the CBAM leads to a reshaped international trade structure and deteriorated global trade conditions, which does a disservice to real income [46] and creates increased inequality. Exports shrink in most countries (Fig. 3). India, Russia, Ukraine, South Africa, and Saudi Arabia are the major regions enduring hits to their exports, with exports declining from 0.17% to 0.36%. In contrast, the EU, EFTA, and UK enjoy the greatest increase by 0.02%–0.1% in exports. The export increase for the EU is due to the increased intra-EU trade rather than exports to the non-EU countries. A 0.02% increase in the Gini coefficient indicates that there is increased inequality through the widening gap of income per capita across countries (Tables 2 and S5). There is a trend toward stronger international trade amongst the developing countries (developing-developing trade). The CBAM improve the comparativeness of the EU's domestically produced goods to some extent and imports from non-EU countries dramatically decrease while trade within the EU is boosted significantly. The CBAM not only gives the EU products a comparative advantage in the EU market, but also weakens the comparativeness of the products in developing countries in the world market while strengthening the comparativeness of the products in the developed countries. This further leads to increased exports from the developed countries to all non-EU countries. As imports from developing countries to developed countries are decreased, the developing countries seek stronger trade links between the developing countries to compensate for the export loss. Therefore, the CBAM witnesses the trend of stronger trade between developing countries.

**Fig. 4:**
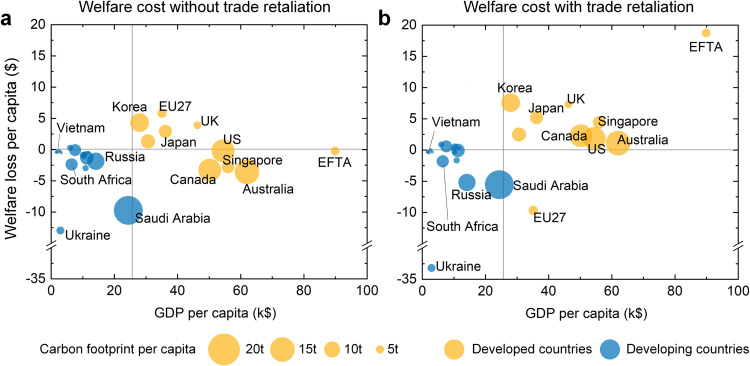
**Economic costs for different income groups.** (a) Welfare costs for different income groups under scenario D90 without trade retaliation. (b) Welfare costs for different income groups under scenario Retaliation with trade retaliation. Countries are classified as developed or developing according to the World Economic Outlook database by International Monetary Fund [41]. Carbon footprint per capita is calculated based on national consumption-based carbon emission from the Global Carbon Budget [43] and population from the GTAP database and the United Nations [47].

Four scenarios regarding different policy schemes are simulated based on D90: 1) the embodied emission accounting method (indirect emissions in the production process, i.e., scope 1 and 2 emissions) is adopted in scenario E90; 2) a higher carbon price of $200/t is applied in scenario D200; 3) carbon intensity based on the EU domestic production technology is applied in scenario D90_EUints; and 4) the CBAM revenue is used to reduce output tax to compensate the price increase due to the CBAM in the EU in scenario D90_otax.

Firstly, consideration of both direct emissions and indirect emissions due to electricity usage contained in import products enhances the effectiveness of the CBAM scheme while adding to the pressure on developing countries. In scenario E90, the carbon emission reduction in non-EU regions is 29.7 Mt, increased by 32% compared with the direct emission accounting in scenario D90 (Table 2). In addition, the negative impacts of the welfare loss increased by 16%, indicating enhanced efficiency of the policy scheme by including indirect emissions in the CBAM. China is the country most negatively impacted by the E90 scheme. The welfare loss rises by almost sevenfold from $1.1 billion to $7.5 billion. Metal products suffer most in China due to the E90 scheme, with the production shrinkage increased from 0.002% to 0.12%. Scope 2 emissions of metal products account for 70% of total scope 1 and scope 2 emissions. China is at a disadvantage because the carbon intensity of power generation in China is amongst the highest in the world, at more than twice that of the EU. The embodied emission scheme could hopefully improve the motivation to decarbonize power generation in the world.

Secondly, higher carbon prices could lift carbon reduction globally and put more pressure on carbon-intensive products outside the EU, but the effectiveness of the CBAM is weakened. Assuming the carbon price will rise to $200/t, the price changes in the EU ETS lead to an extra carbon reduction by 372.9 Mt CO_2_ emissions. And this further leads to 108.9 Mt carbon leakage, causing an enlarged carbon leakage rate of the EU ETS from 20.2% to 23.7%. At the same time, carbon emissions reduced outside the EU by the CBAM are 36.4 Mt, with 8.1 Mt rebounded in the EU and exempted areas. Although the reduced carbon emissions are enhanced by higher carbon prices, the CBAM shows lessened capacity to address carbon leakage problems. The reduced carbon emissions outside the EU accounts for 16% of the total emission rise due to leakage, which is lower than the leakage reduction rate in D90.

Thirdly, the scenario D90_EUintst uses the carbon intensity of the EU production process to calculate tariff changes. This scheme helps to simplify the policy implementation but reduces the effect of the CBAM. The carbon emission reduction by the CBAM in non-EU regions only accounts for 8.8% of the EU ETS carbon leakage, while the economic impact is slightly changed. The carbon emissions per unit production in the EU, especially of the carbon-intensive products, are amongst the lowest globally due to the ambitions of the EU in decarbonizing and mitigating climate change. Therefore, using the EU technology for measuring carbon content of the imports significantly relieves the pressure that the non-EU countries experienced, but also reduce the effectiveness of the CBAM and weakens the decarbonization motivation of producers outside the EU.

Fourthly, revenue recycling plays an important role in protecting the EU regarding the macroeconomic impacts of the CBAM. With the CBAM revenue used to reduce output tax in the EU, the environmental benefits are only slightly affected but the economic cost is considerably reduced. However, it is worth noting that such welfare loss reduction is not because of the overall benefits of the global nations but exclusively enjoyed by the EU. By recycling the CBAM revenue, the welfare gains of the EU increased by 40%, while other regions experience more welfare loss. Detailed simulation results in these scenarios can be found in Figs. S3–8.

### 3.2 Trade retaliation multiplies economic costs and increases inequality

The scale of trade conflicts includes the countries taking countermeasures, the products targeted by the trade conflicts, and the range of tariff changes. Here, we designed a scenario to simulate the impacts of trade retaliation based on scenario D90. Under scenario Retaliation, the EU's major trade partners impose an extra tariff on EU products that are often the objects of anti-dumping measures, and the retaliatory tariff is determined by export losses the raiser experiences.

The results imply that trade retaliation against the CBAM has limited impacts on the carbon reduction achievement of the CBAM but leaves the world more vulnerable to the economic loss and inequality in climate change mitigation. The carbon reduction in non-EU regions amounts to 19.6 Mt under the scenario Retaliation, which is less than the emission reduction when there is no trade retaliation. Retaliatory tariffs on EU productions increase trade within the non-EU countries and thus offset the carbon emissions reduction via the CBAM. On the other hand, economic losses are multiplied by trade retaliation. Under the scenario Retaliation, the EU, Ukraine, and Saudi Arabia experience the most GDP losses, ranging from 0.1% to 1.5%. The welfare loss amounts to 4.6 billion dollars, which is nearly seven times the welfare loss under scenario D90. The EU suffers the most welfare losses of $4.3 billion. International trade conditions are unambiguously deteriorated, with an expansion of export losses. Most countries experience risen prices of domestic production and therefore exports. For example, chemistry and plastic are amongst the most impacted sectors in South Africa, with prices edged up by 0.5% and 0.3% domestically. Consequently, exports in India, South Africa, and Russia decrease by 0.6%, 0.5% and 0.4%, respectively.

The welfare losses of retaliating against the CBAM and the corresponding trade barriers between countries leave poor countries more vulnerable. As trade retaliation causes welfare losses or gains for different countries, the wealthy countries as a group enjoy net benefits while the poor countries as a group suffer net losses (Fig. 4). The EFTA countries are the richest and receive welfare gains of $18.8 per capita under scenario Retaliation. On the other hand, people in Ukraine face welfare losses of $33.2 per capita. Clearly, the economic costs of the CBAM and trade retaliation are borne by the poorest group, thereby leaving economically vulnerable people worse off.

### 3.3 The CBAM is less effective than international cooperation

A Cooperation scenario sees significantly enhanced carbon reduction benefits with slightly increased economic costs. In the scenario Cooperation, the floor carbon price is implemented according to the development of the countries. The developed countries impose a carbon price equal to the carbon price in the EU, and high-income emerging countries have a carbon price lower than the EU ETS at $60/t, while the low-income emerging countries have the lowest carbon price at $30/t. The carbon reduction effects of the three types of carbon prices are 3,372.1 Mt in developed countries, 4,187.2 Mt in high-income developing countries, and 850.4 Mt in low-income developing countries. The carbon leakage rates of the floor carbon prices are 0.9%, 3.8% and 4.7% respectively. As the floor carbon prices are implemented, the global carbon leakage is greatly reduced to 0.6%. Comparing the economic loss of carbon prices in different countries, the price in high-income developing countries leads to higher abatement benefits at a lower economic cost. The global welfare and GDP loss caused by the carbon prices in the developed countries is 0.18% and 0.16% respectively. These economic losses are relatively equal with the EU ETS and carbon prices in the developing countries. But the carbon reduction in the high-income developing countries is apparently larger, about 14% of global total carbon emissions.

The CBAM in the scenario Cooperation imposes a tariff on the developing countries to compensate for the carbon price gap between the EU and the developing countries. The results show that the carbon reduction by the CBAM is 10.3 Mt in the non-EU regions, which accounts for 8.6% of the carbon leakage caused by the EU ETS and 46% of the benchmark CBAM scheme in scenario D90. Considering the carbon prices in the developing countries in scenario Cooperation, the effectiveness of the CBAM pales because the carbon reduction only accounts for 0.2% of the carbon reduction by carbon prices in these countries. Overall, floor carbon price can impose carbon abatement measures globally and essentially reduce carbon leakage by acknowledging the differentiated levels of carbon reduction costs according to the degree of economic development. Therefore, compared with the unilateral CBAM scheme, assisting the developing countries to promote their climate change mitigation ambition and address the carbon emission embedded in the domestic production in these countries will lead to far larger environmental benefits to the world's climate change mitigation.

## 4 Discussion and conclusion

Simulations of different internal factors of the CBAM indicate that accounting for embodied emissions and using the carbon intensity of the local production technologies lead to more environmental benefits, and that revenue used to reduce output tax further brings the EU more economic gains. Firstly, embodied emissions accounting opens the opportunity for the CBAM to facilitate the global energy transition and technology upgrading. The indirect emissions of products vary greatly across countries, and in this respect, electricity usage is the most important source [6,34]. Both electricity usage in production, and the carbon intensity of electricity generation, are different due to the technology factors in different countries [48]. Therefore, countries that rely more on high carbon-intensive electricity, such as China and India, suffer more from the CBAM applying embodied emission accounting. In contrast, countries with cleaner energy generation will gain comparative advantages from such a scheme. For instance, Brazil enjoys a GDP increase because of its energy structure. In 2019, non-fossil energy in Brazil accounted for 45.7% of its total energy consumption, which is amongst the highest in the world [49]. Secondly, measuring the carbon intensity of the production process in the original export country improves the effectiveness of the CBAM. As it is designed, the CBAM needs to compensate for the carbon abatement cost of the EU's domestic production and improve the comparative advantages of the EU's products. Measuring the carbon content of imported goods according to the average level in the origins reflects the technology level of the production process and therefore improves the motivation of the producers to decarbonize their production process. The CBAM, if based on the average carbon intensity of EU producers rather than the actual carbon content of imports, could of course reduce the complication and cost of policy implementation. But it is inadequate to compensate the carbon abatement cost of the EU's domestic producers and thus shows less efficiency in addressing carbon leakage. Thirdly, the CBAM revenue, recycled to reduce the output tax and compensate for the increased price in the EU's domestic market, further deteriorates the inequality conditions caused by the CBAM. With reduced output tax, the EU has a significant increase in welfare gains, but other countries bear an extra welfare loss. Considering the macroeconomic impact of the revenue recycling, there is an increased legal risk that the CBAM may be recognized as domestic protectionism and a resource for the EU's own fiscal budget.

Although the environmental benefits of the CBAM can be enlarged with appropriate scheme settings, the overall carbon leakage reduction effect is rather limited, and the higher carbon price brings with it even less effectiveness of the CBAM to address carbon leakage problems. The CBAM scheme at the current carbon price in the EU ETS can reduce emissions outside the EU by only 10.5–29.7 Mt, accounting for a small proportion of the emission leakage caused by the EU ETS (8.8%−24.9%). Worse still, the effectiveness of the CBAM to address the carbon leakage risks of the EU ETS is weakened under a higher carbon price, leading to greater economic burden as well as risks of shrinking the market size of the EU's products [50]. To improve compatibility with GATT actions, it is necessary to constrain the carbon price in the CBAM so as not to exceed the price level in the EU ETS. As carbon quotas tighten and carbon prices rise, the carbon leakage ratio is enlarged in the EU ETS in the future, but the CBAM shows less capability to address such leakage. The carbon reduction by the CBAM accounts for a lower proportion of the total carbon leakage (from 18.9% to 16%). Therefore, the increased ambition of mitigating climate change in the EU needs to be accompanied with further measurements to boost climate ambition outside the EU as the unilateral competitive policy scheme shows less capacity in dealing with such imbalanced climate efforts.

Regarding the weak carbon leakage reduction effect, one reason is that the CBAM cannot fully compensate for the competitiveness losses that domestic firms face because the carbon tariff on the imports is relatively small. Therefore, the changes in the price are slight. For example, the ad valorem tariff rates by the CBAM on chemical products and mineral products from China are 4.3% and 6.6%, according to indirect emission accounting (supplementary data and Fig. S2), respectively. Other manufacturing sectors and regions usually impose even lower carbon tariffs. Consequently, the affected trade volume is narrow because the change in demand is small. In other words, the competitive advantages of EU and non-EU carbon-intensive products change slightly, and thus, the effect of global industrial restructuring is limited.

Another reason is related to the low level of price elasticities for intermediate inputs. In the EU, 80.7% of total imports are purchased by firms as intermediate inputs for domestic production, while the other 19.3% of total imports are consumed by government and private households. In addition, the import proportion of the carbon-intensive products purchased by the EU firms is higher than other products. Specifically, in 2014, 18.5% of their carbon-intensive intermediate input products were imported, while only 11.1% of the non-carbon-intensive inputs were imported. As intermediate input purchases are less sensitive to price than final consumption, there are innate defects in the CBAM mechanism regardless of its design. The increasing carbon prices in the future could expand the environmental benefits, but they will also cause adverse impacts on EU firms by increasing their production costs and contracting their market size [48].

Last but not least, the essential deficiency of the CBAM is that it fails to address carbon leakage happening in the global energy market. Due to the very nature of the mechanism of the CBAM, it mainly improves competitiveness of domestic carbon-intensive products by raising the price of import products, which is one of three patterns of carbon leakage. The second is the international relocation of carbon-intensive industries. Firms tend to transfer their carbon-intensive production to non-abatement countries to avoid the environmental costs in abatement countries. Finally, carbon leakage happening in the global energy market is also very important [51], especially for large emissions abatement groups [3]. Carbon reduction measures cause decreases in the demand for fossil fuel in abatement countries and further lead to decreases in the energy price in the global market. These decreases adversely stimulate the energy demand of non-abatement countries and increases their carbon emissions. The CBAM does not limit this last type of carbon leakage because it has little impact on reducing non-EU countries’ domestic demand for fossil fuel energy [50,^52^,53]. Additional measures to restrict fossil fuel supply may assist in tackling this problem [54].

If other countries initiate trade retaliation in response to the CBAM, concerns of inequality will be enlarged. Through the CBAM, the EU enjoys the most economic gains. In addition to the EU, other major developed countries gain competitive advantages by carbon-based tariffs and obtain increased demand for carbon-intensive products. In contrast, developing countries usually experience a reduction in total production due to declined market share, and have the most welfare losses. Consequently, the gap between developing countries and developed countries is further widened and global Gini coefficient is enlarged. Trade retaliation, justified by the export and production losses, leads to an offset of the CBAM, with reduced environmental benefits but multiplied economic losses. As a result, the EU would be faced with a significant loss of real income and domestic production because it is a net exporter of carbon-intensive goods and would be sensitive to trade barriers in these sectors. However, retaliation still fails to compensate for the losses of developing countries. Even worse, the inequality is magnified as poorest countries bear the most economic costs while the richest enjoy the most benefits.

In the light of the ineffectiveness of the CBAM as a unilateral measure, in reducing carbon leakage and the aggravated inequality, we suggest that international cooperation on technological innovation is vital for global climate change mitigation. As is officially stated in the proposal of the CBAM, strong international cooperation will strengthen the joint climate action [14]. The signal effect of the CBAM may precipitate the establishment and improvement of carbon pricing in non-EU countries and therefore promote global climate action. There is no denying that the intention of the CBAM to address carbon leakage is in accordance with the EU's climate ambition and its domestic efforts. However, evasion measures may also be taken by EU trade partners, for example, inaccurate emissions report or symbolic policy. The intensified global competition resulting from the CBAM may lead to a detrimental environment for international trust, making sustainable development with equality and climate change mitigation far more costly. Overall, climate change mitigation and global carbon reduction should be based on a thorough transition of the economic structure and energy mix. Technology upgrading and green development are critical in this process [55,56]. Developing countries have an urgent need for access to green technologies and the necessary financial support. However, the CBAM scheme is a solution based on competitiveness protection, and it neglects technology transfer. We suggest that major economies should pursue the chance to promote international climate cooperation and enable developing countries to access green technologies. For example, the floor carbon prices in this study are effective in addressing the carbon leakage of the abatement countries. In addition, improving the motivation of more ambitious carbon pricing in developing countries is efficient with less economic costs but more emission reduction gains.

## Declaration of competing interest

The authors declare that they have no conflicts of interest in this work.

## Appendix Supplementary materials

Image, application 1

Image, application 2
