# Supplementary material for: The carbon border adjustment mechanism is inefficient in addressing carbon leakage and results in unfair welfare losses
Source: Fundam Res. 2023 Mar 30;4(3):660–70. doi: 10.1016/j.fmre.2023.02.026 (PMC11630705; doi:10.1016/j.fmre.2023.02.026)
Supplement: Supplementary file 1 [file mmc1.docx]

# Supplementary materials

# The Carbon Border Adjustment Mechanism is inefficient in addressing carbon leakage and results in unfair welfare losses

Xinlu Sun, Zhifu Mi^*^, Lu Cheng, D’Maris Coffman, Yu Liu

**Sensitivity analysis for substitution parameters**

We perform a sensitivity analysis to confirm the robustness of the results in this study, as CGE models receive concerns for the sensitivity to changes in parameters. Elasticity parameters in trade and production are the most important parameters in affecting the overall results because they determine the degree of the producers’ and consumers’ respond to a policy or economic shock [1,2]. We apply the Armington elasticity from Németh et al. (2011) [1] for domestic/import substitution as well as import substitution among different exports (scenarios with suffix EARM). We also apply the substitution elasticity of capital-energy composite from Beckman et al. (2011) [3] to test the impact of these parameters on our results (scenarios with suffix EFKE). Both types of parameters are tested in scenarios with suffix ESUB. Sensitivity analysis with changes in elasticity parameters is first applied for the ETS of all countries, and the results of EU ETS are shown. Then, sensitivity analysis is performed for the CBAM under the benchmark scenario D90.

Overall, the sensitivity analysis confirms the robustness of our results (Table S6). Different parameters indicate the carbon leakage rates in the EU ETS are among 16.8-20.4%, and the CBAM D90 can reduce carbon leakage by 15.6-22.8%. Changing the Armington elasticity in this study leads to less carbon leakage because smaller Armington elasticity indicates the domestic producers in EU are less sensitive to the rise in domestic price compared with imported goods, and therefore less carbon leakage. Regarding the effectiveness of CBAM, EU domestic producers are not very sensitive to the increased costs of using imported carbon-intensive inputs with smaller Armington elasticity and therefore it weakens the mitigation of carbon leakage by the CBAM. With larger elasticity in the substitution of capital-energy composite in the original GTAP-E model, the estimated carbon reduction in EU ETS is larger compared with the results of sensitivity analysis. This is because larger capital-energy substitution makes the producer more sensitive to increased cost in energy inputs and therefore shift inputs from energy to capital. But the effectiveness of carbon reduction in EU ETS does not change much and CBAM could reduce carbon leakage to a larger degree. As carbon abatement leads to drop in energy prince internationally, developing countries without any abatement measures tend to prefer energy inputs which offsets their efforts in responding to the CBAM. Smaller substitution elasticity of capital-energy composite indicates less preference in energy inputs considering a price decrease of energy products in developing countries and thus leads to more effective CBAM.

| 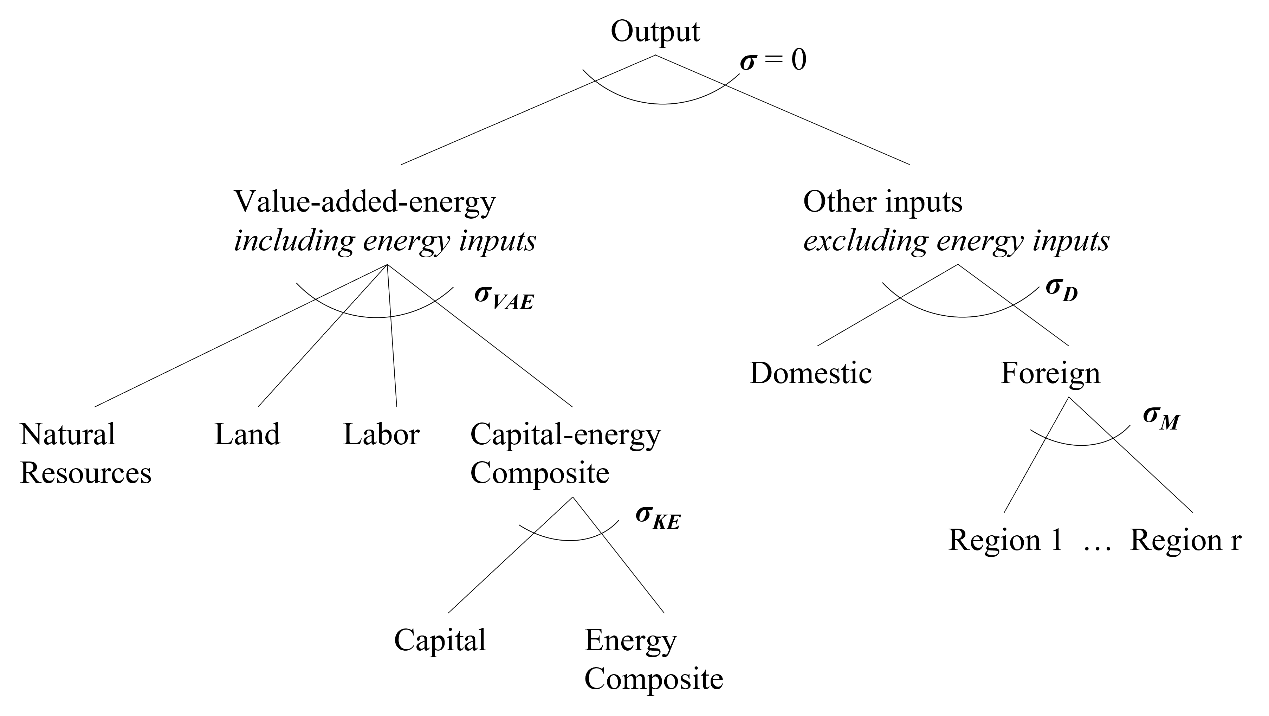 |
| --- |
| Fig. S1. Production structure in the GTAP-E model |

| 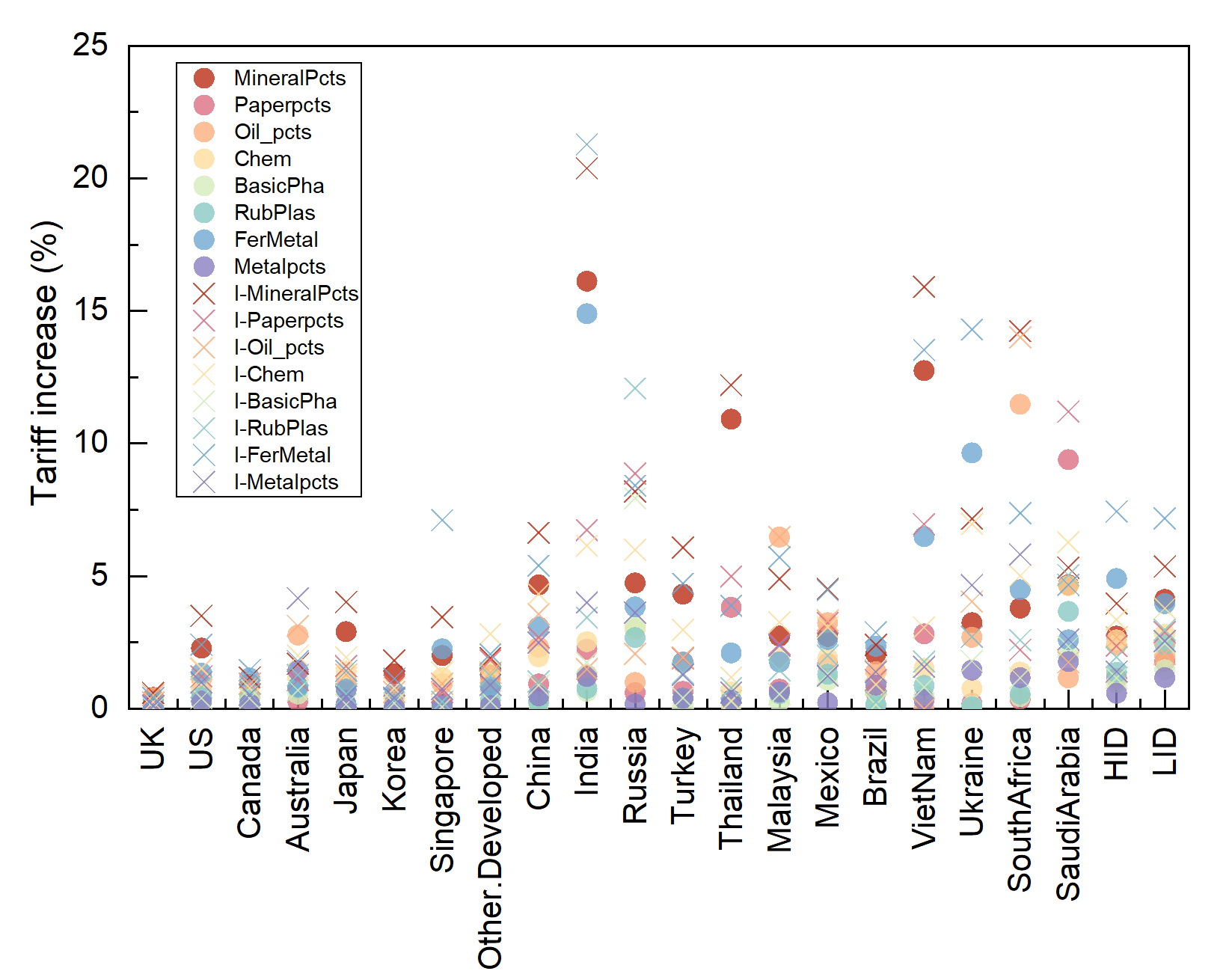 |
| --- |
| Fig. S2. Tariff changes in scenario D90 and E90. Sectors denoted by dots means tariff changes due to the CBAM D90 and only direct emission (scope 1) are measured. Sectors with a prefix “I-” means tariff changes due to the CBAM E90, where indirect emissions (scope 1 and 2) embedded in the products are accounted for. |

| 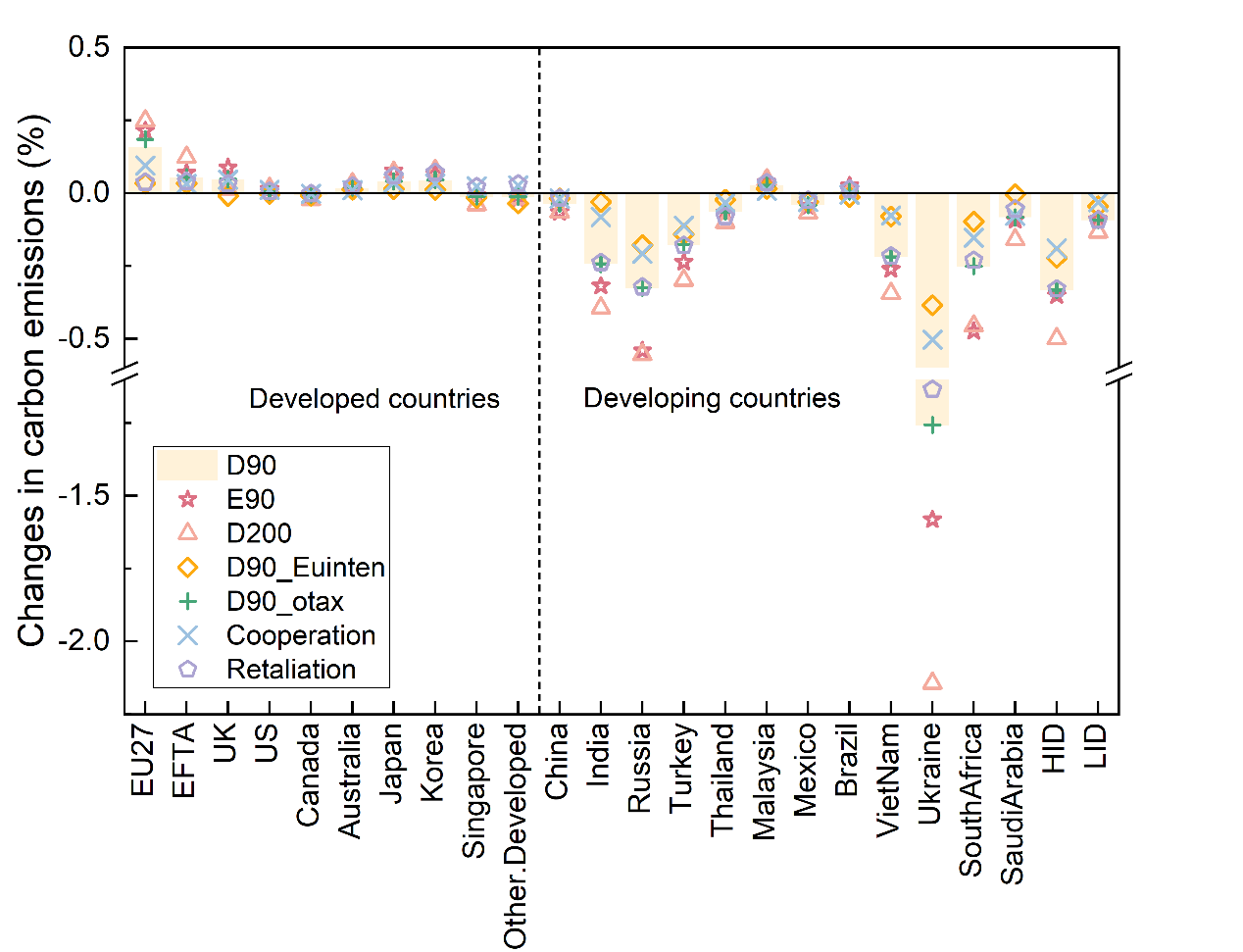 |
| --- |
| Fig. S3. Carbon emission changes in different scenarios. |

| 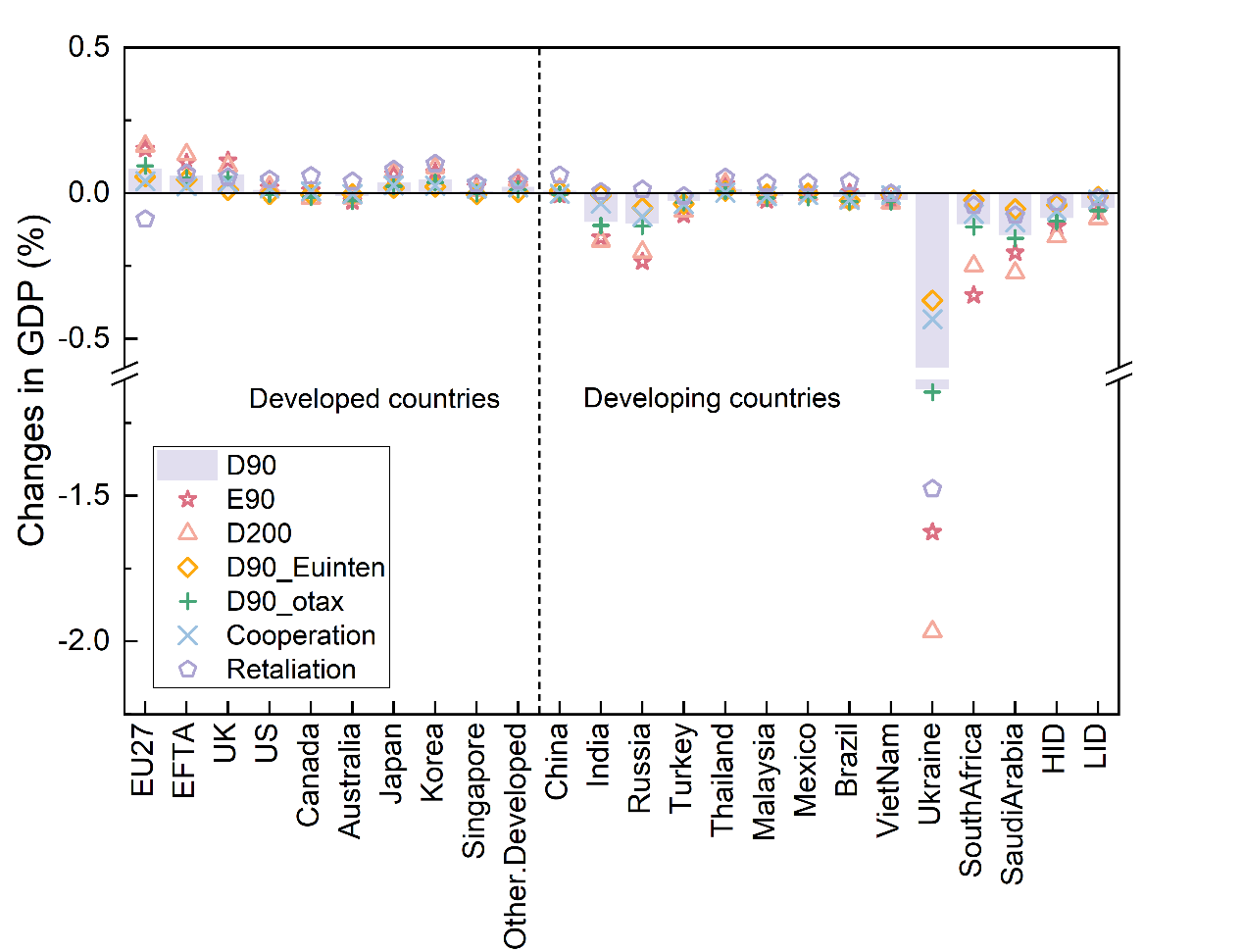 |
| --- |
| Fig. S4. GDP changes in different scenarios. |

| 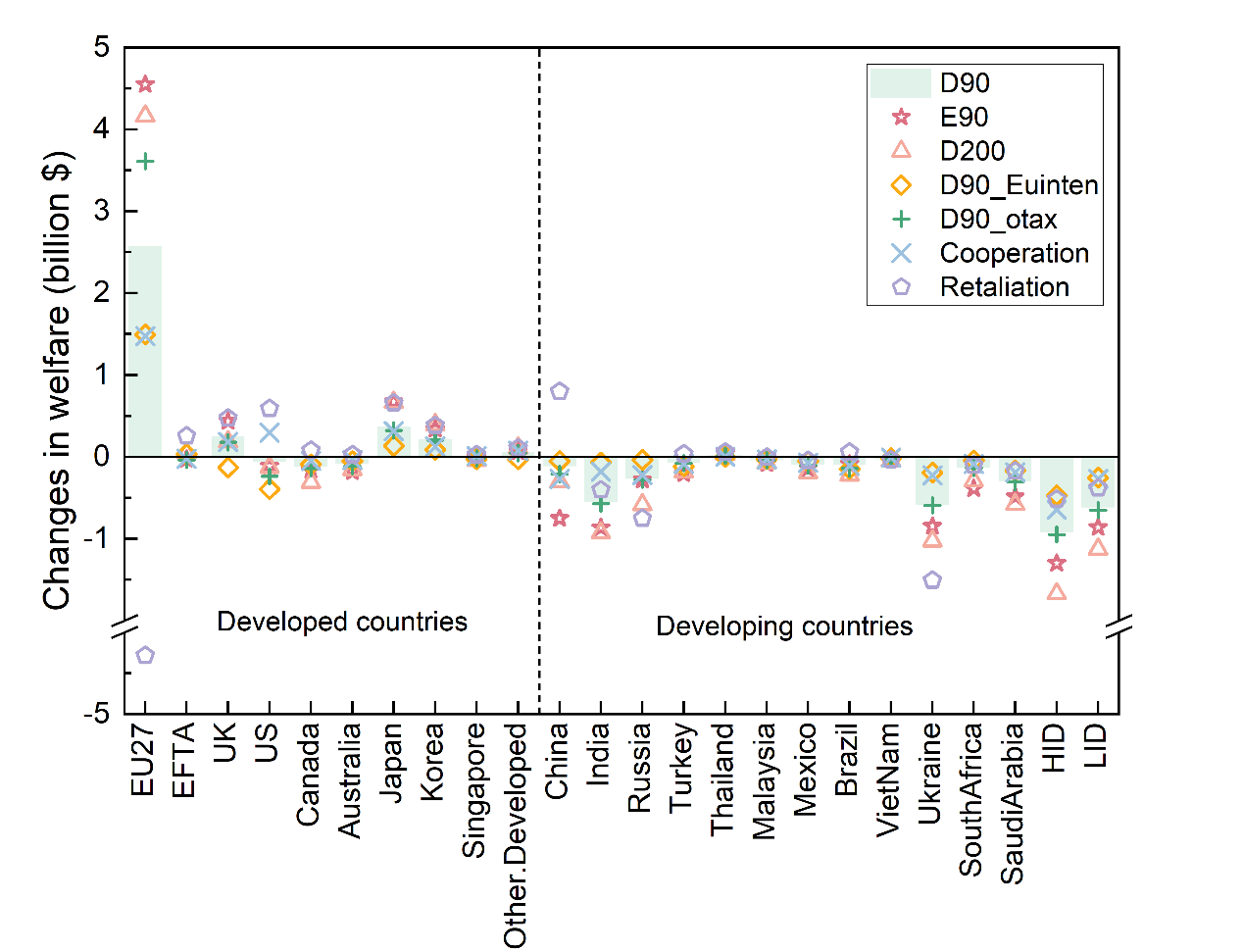 |
| --- |
| Fig. 5. Welfare changes in different scenarios. |

| 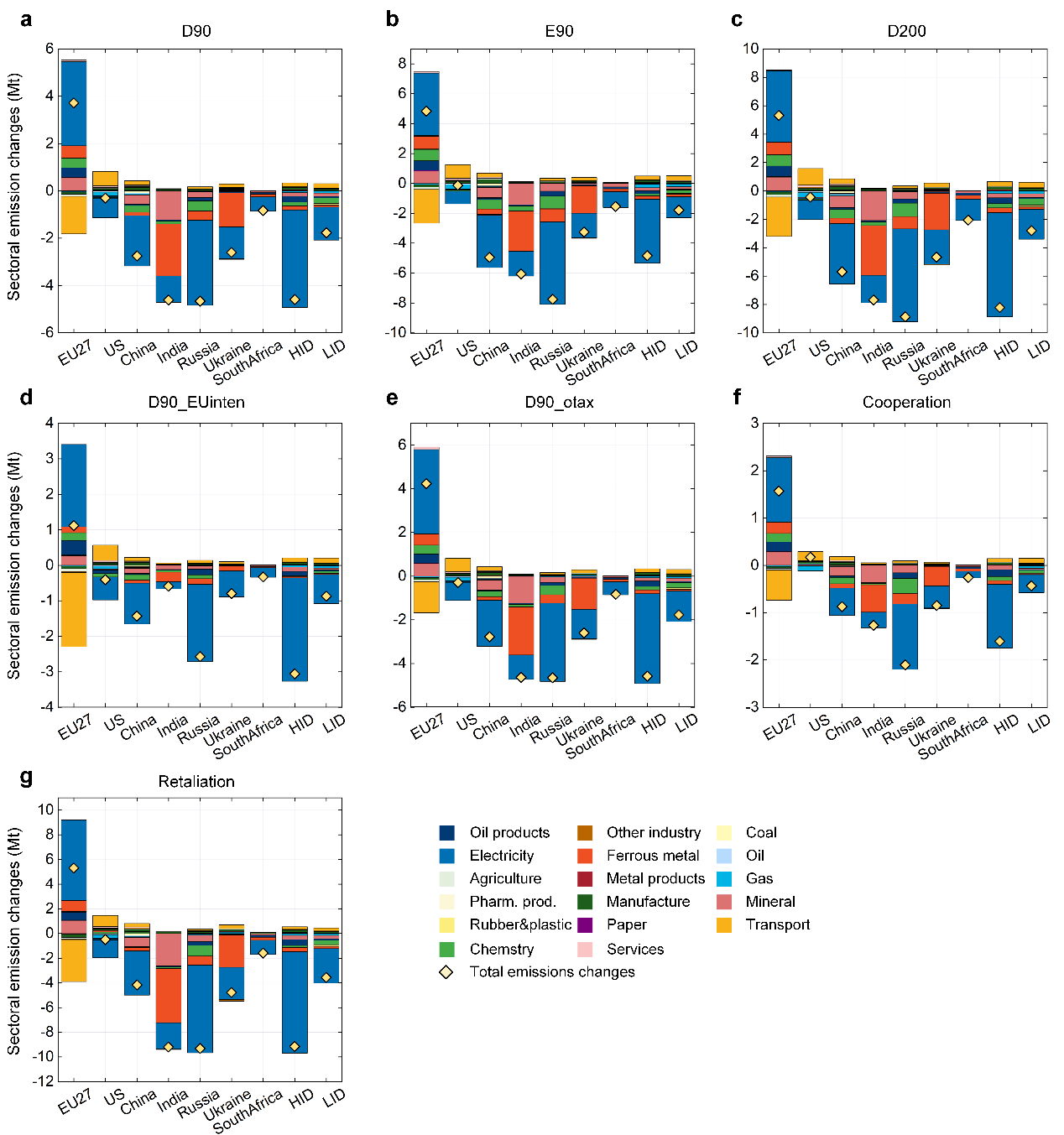 |
| --- |
| Fig. S6. Sectoral emission changes of major impacted countries in different scenarios. |

| 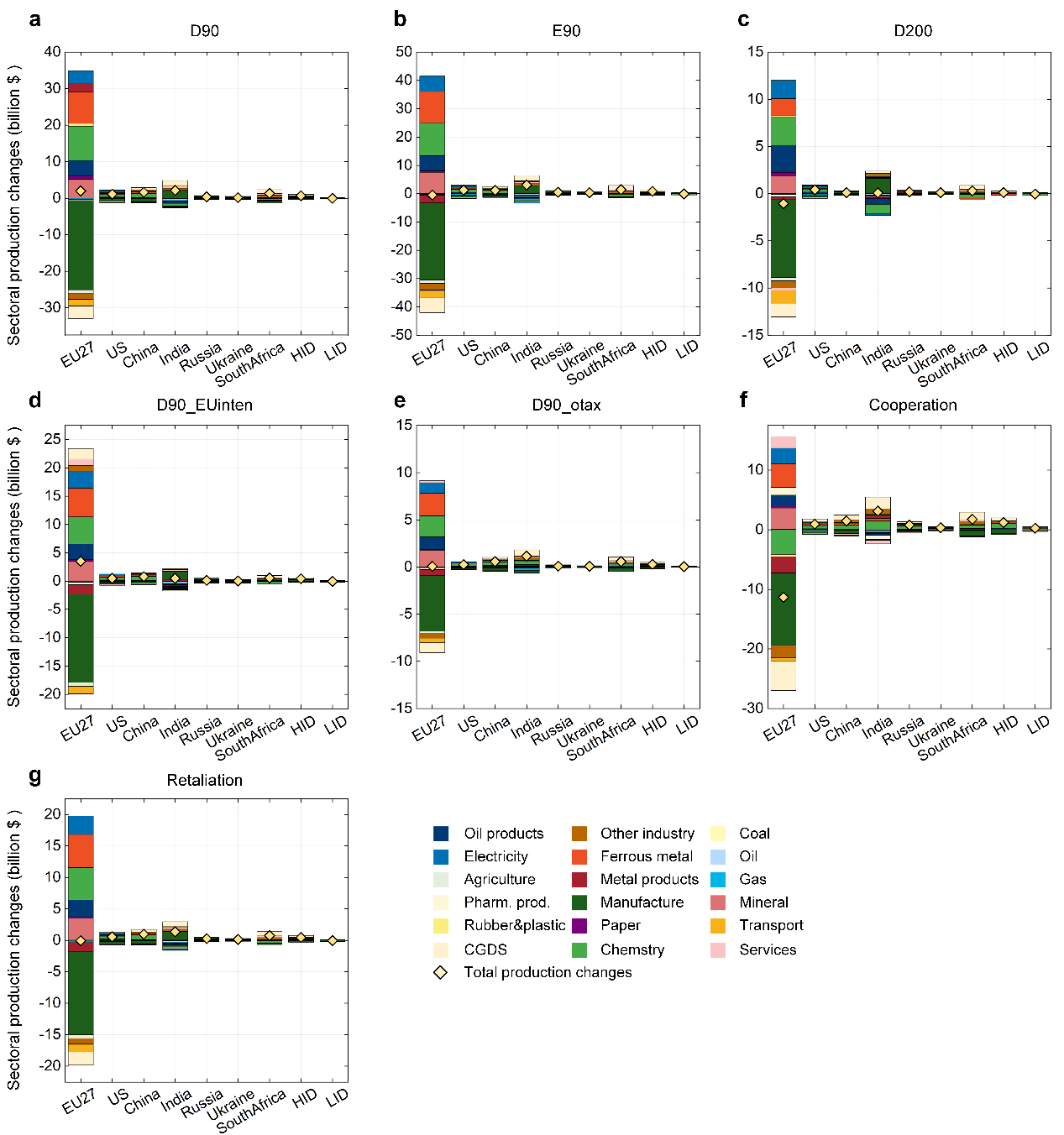 |
| --- |
| Fig. S7. Sectoral production changes of major impacted countries in different scenarios. |

| 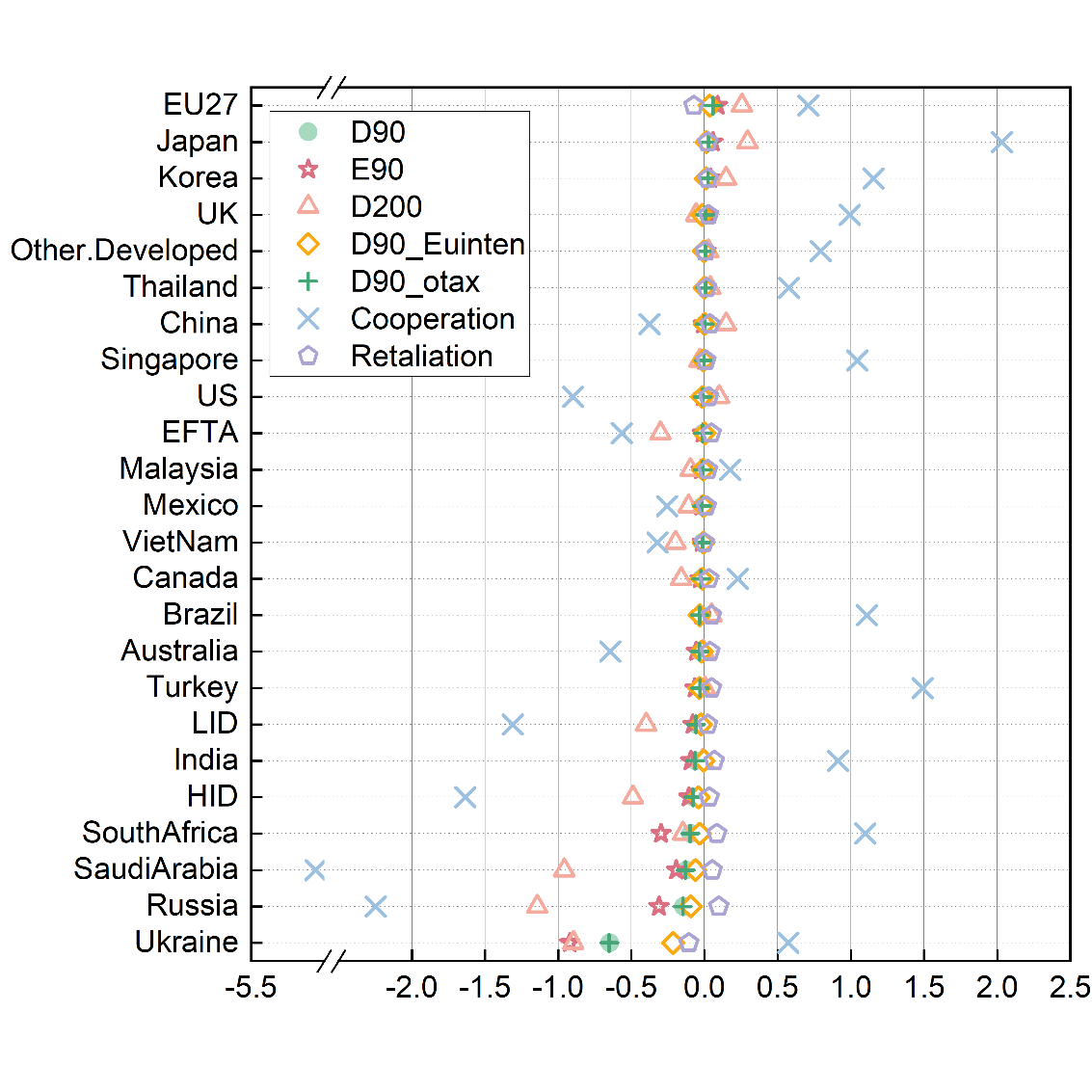 |
| --- |
| Fig. S8. Changes in terms of trade (tot) in different scenarios. |

Table S1. Regions and sectors in the CGE model

| **Region codes** | **Region** | **Sector codes** | **Sectors** |
| --- | --- | --- | --- |
| EU27 | EU27 | **Carbon-intensive sectors (excluding energy sectors)** | |
| EFTA | European Free Trade Association countries | MineralPcts | Mineral products |
| UK | United Kingdom | Paperpcts | Paper products |
| US | United States | Chem | Chemistry |
| Canada | Canada | BasicPha | Basic pharmaceutical products |
| Australia | Australia | RubPlas | Rubber and plastic products |
| Japan | Japan | FerMetal | Ferrous metal |
| Korea | Korea | Metalpcts | Metal products |
| Singapore | Singapore |  |  |
| Other.Developed | Other developed countries | **Energy** |  |
| China | China | Coal | Coal |
| India | India | Oil | Oil |
| Russia | Russia | Gas | Gas |
| Turkey | Turkey | Oil_pcts | Oil products |
| Thailand | Thailand | Electricity | Electricity |
| Malaysia | Malaysia |  |  |
| Mexico | Mexico | **Other sectors** | |
| Brazil | Brazil | Agric | Agriculture |
| VietNam | VietNam | Manufac | Manufacture |
| Ukraine | Ukraine | Otherindus | Other industries |
| SouthAfrica | South Africa | Services | Services |
| SaudiArabia | Saudi Arabia | Transport | Transport |
| HID | Higher-income developing countries |  |  |
| LID | Lower-income developing countries |  |  |
| LDC | Less developed countries |  |  |
| ROW | Rest of world |  |  |

Note: Higher-income developing countries include Brunei Darussalam, Argentina, Chile, Colombia, Ecuador, Paraguay, Peru, Uruguay, Costa Rica, Guatemala, Panama, Dominican Republic, Jamaica, Puerto Rico, Trinidad and Tobago, Albania, Belarus, Kazakhstan, Armenia, Azerbaijan, Georgia, Bahrain, Jordan, Kuwait, Oman, Qatar, United Arab Emirates, Cote d'Ivoire, Botswana, Namibia; Lower-income developing countries include Mongolia, Indonesia, Philippines, Pakistan, Sri Lanka, Bolivia, Venezuela, Honduras, Nicaragua, El Salvador, Caribbean, Kyrgyzstan, Tajikistan, Iran, Egypt, Morocco, Tunisia, Cameroon, Ghana, Nigeria, Senegal, South Central Africa, Kenya, Zimbabwe. Countries are grouped according to World Band Country and Lending Groups ([World Bank Country and Lending Groups – World Bank Data Help Desk](https://datahelpdesk.worldbank.org/knowledgebase/articles/906519-world-bank-country-and-lending-groups)).

Table S2. Current carbon prices in the world implemented in the scenario ETS.

| Region | Carbon price | Covered emissions | Coverage rate |
| --- | --- | --- | --- |
|  | $/t | Mt | % |
| EU27 and EFTA | 90.33 | 1725.77 | 0.42 |
| UK | 91 | 134.12 | 0.29 |
| US | 14.86 | 524.79 | 0.08 |
| Canada | 29.92 | 480.2 | 0.63 |
| Japan | 2.66 | 1029.53 | 0.81 |
| Korea | 28.97 | 513.42 | 0.68 |
| Singapore | 3.71 | 44.78 | 0.64 |
| China | 7.16 | 4647.79 | 0.34 |
| Mexico | 3.18 | 188.19 | 0.23 |
| Ukraine | 0.36 | 221.58 | 0.80 |
| South Africa | 9.15 | 512.25 | 0.89 |

Note: For the main emission trading markets where carbon price kept increasing in 2021 and remain high in 2022, carbon price in the last month of 2021 is implemented, e.g., EU and UK. For other countries, the average carbon prices in 2021 are implemented. The prices were deflated to remove the inflation influence before applied in the GTAP model.

Table S3. Regions with exemptions

| **Region codes** | **Regions in the GTAP database** |
| --- | --- |
| EFTA | Switzerland |
|  | Norway |
|  | Rest of EFTA |
| LDC | Cambodia |
|  | Lao People's Democratic Republic |
|  | Bangladesh |
|  | Nepal |
|  | Benin |
|  | Burkina Faso |
|  | Guinea |
|  | Togo |
|  | Central Africa |
|  | Ethiopia |
|  | Madagascar |
|  | Malawi |
|  | Mauritius |
|  | Mozambique |
|  | Rwanda |
|  | Tanzania |
|  | Uganda |
|  | Zambia |
| ROW | Rest of Oceania |
|  | Rest of East Asia |
|  | Rest of Southeast Asia |
|  | Rest of South Asia |
|  | Rest of North America |
|  | Rest of South America |
|  | Rest of Central America |
|  | Rest of Eastern Europe |
|  | Rest of Europe |
|  | Rest of Former Soviet Union |
|  | Rest of Western Asia |
|  | Rest of North Africa |
|  | Rest of Western Africa |
|  | Rest of Eastern Africa |
|  | Rest of South African Customs |
|  | Rest of the World |

Table S4. Major trade partner of the EU and target sectors in bilateral trade disputes

| **Trade partner** | **Target sectors in trade disputes** | | |
| --- | --- | --- | --- |
| USA | Ferrous metal | Metal products |  |
|  | Chemistry |  |  |
| Canada | Ferrous metal | Metal products |  |
|  | Manufacture |  |  |
| Australia | Chemistry | Rubber and plastic products |  |
| Japan | Chemistry |  |  |
| Korea | Chemistry | Manufacture |  |
| China | Chemistry | Rubber and plastic products |  |
| India | Chemistry | Manufacture |  |
| Russia | Manufacture | Ferrous metal |  |
|  | Metal products |  |  |
| Turkey | Ferrous metal | Rubber and plastic products |  |
|  | Metal products |  |  |
| Thailand | Ferrous metal | Metal products |  |
| Malaysia | Paper products | Ferrous metal |  |
|  | Metal products |  |  |
| Mexico | Chemistry | Ferrous metal |  |
|  | Metal products |  |  |
| Brazil | Chemistry | Ferrous metal |  |
|  | Metal products |  |  |
| Ukraine | Mineral products | Manufacture |  |
| South Africa | Paper products | Chemistry |  |

Table S5. Gini coefficient and carbon emission coefficient in different scenarios.

|  | **Gini coefficient** | **Gini coefficient change (%)** | **C-Gini coefficient** | **C-Gini coefficient change (%)** |
| --- | --- | --- | --- | --- |
| **ETS. At current carbon prices** | **0.60357** |  | **0.44978** |  |
| D90 | 0.60360 | 0.004 | 0.44997 | 0.019 |
| E90 | 0.60363 | 0.006 | 0.44998 | 0.019 |
| D90_Euinten | 0.60358 | 0.001 | 0.44981 | 0.002 |
| D90_otax | 0.60361 | 0.004 | 0.44997 | 0.019 |
| Retaliation | 0.60360 | 0.003 | 0.4500 | 0.022 |
| **D200. EU ETS at 200$/t** | **0.60336** |  | **0.45159** |  |
| D200 | 0.60342 | 0.006 | 0.45182 | 0.022 |
| **Cooperation. Floor carbon prices** | **0.60407** |  | **0.43177** |  |
| Cooperation | 0.60409 | 0.002 | 0.43189 | 0.012 |

Table S6. Sensitivity analysis for substitution elasticity parameters.

|  | **CO_2_** | **Emission leakage** | **Leakage rate** | **Welfare** |
| --- | --- | --- | --- | --- |
|  | **Mt** | **Mt** | **%** | **Billion$** |
| **EU ETS. At current carbon prices** | | | | |
| EUETS | -588.9 | 119.1 | 20.2 | -86.2 |
| EUETS_EFKE | -510.0 | 103.9 | 20.4 | -77.3 |
| EUETS_EARM | -564.0 | 102.8 | 18.3 | -83.9 |
| EUETS_ESUB | -485.9 | 81.8 | 16.8 | -76.5 |
|  | **CO_2_ in non-EU** | **Emission leakage reduction** | **CO_2_ in EU and exempted areas** | **Welfare** |
|  | **Mt** | **%** | **Mt** | **Billion$** |
| **ETS+CBAM. At current carbon prices** | | | | |
| D90 | -22.5 | 18.9 | 4.9 | -0.67 |
| D90_EFKE | -23.7 | 22.8 | 6.5 | -0.61 |
| D90_EARM | -16.1 | 15.6 | 3.9 | -0.76 |
| D90_ESUB | -16.7 | 20.4 | 4.2 | -0.76 |

Note: EU ETS means the economic and climate impacts of carbon prices in EU ETS. ETS+CBAM means the economic and climate impacts of the CBAM taking current prices in all countries as the baseline scenario. EFKE represents replacing elasticity parameters in substitution of capital-energy composite. EARM represents replacing Armington elasticity parameters. ESUB represents replacing both Armington elasticity parameters and elasticity parameters in substitution of capital-energy composite.

**Supplementary References**

[1] Németh G, Szabó L, Ciscar J-C. Estimation of Armington elasticities in a CGE economy–energy–environment model for Europe. Economic Modelling 2011;28:1993–9. https://doi.org/10.1016/j.econmod.2011.03.032.

[2] Antimiani A, Costantini V, Martini C, Salvatici L, Tommasino MC. Assessing alternative solutions to carbon leakage. Energy Econ 2013;36:299–311. https://doi.org/10.1016/j.eneco.2012.08.042.

[3] Beckman J, Hertel T, Tyner W. Validating energy-oriented CGE models. Energy Economics 2011;33:799–806. https://doi.org/10.1016/j.eneco.2011.01.005.
